# Europium-doped cerium oxide nanoparticle-impregnated Hyalgan® (CartiOxgel): an intra-articular contrast agent for X-ray CT and optical imaging†

**DOI:** 10.1039/d5ra01830g

**Published:** 2025-07-07

**Authors:** Hema Brindha Masanam, Sina Jafari, Ashwin Kumar Narasimhan, Priyatha Premanth, Margaret Salomi, Victor R. Lazar, Sentil Kumar Aiyappan, Sruthi Ann Alex

**Affiliations:** a Department of Biomedical Engineering, SRM Institute of Science and Technology Kattankulathur-603203 Tamil Nadu India sruthia@srmist.edu.in; b Department of Biomedical Engineering, College of Engineering and Applied Science, University of Wisconsin 3200 North Cramer Street Milwaukee WI 53211 USA; c Specific Pathogen Free Animal House (SPF-AH), SRM Institute of Science and Technology Kattankulathur-603203 Tamil Nadu India; d Department of Radio-Diagnosis, SRM Medical College Hospital and Research Centre Kattankulathur-603203 Tamil Nadu India

## Abstract

Cartilage internalizing metal-oxide gel (“CartiOxgel”), a novel hybrid contrast agent, was developed for enhanced X-ray computed tomography (CT) and optical intra-articular (IA) imaging. The formulation comprises europium (Eu)-doped cerium oxide nanoparticles (CeO_2−*x*_ NPs, C), denoted as Eu_*x*%_Cy NPs, which were incorporated into Hyalgan®, a commercial intra-articular (IA) injectable gel. Eu doping at 2% and 10% was achieved using a one-pot combustion method (as-prepared at 200 °C, *y* = 2), followed by thermal treatment at 700 °C (*y* = 7) to investigate the effect of heat treatment on hybrid NP formation. Physicochemical characterization confirmed the structural stability and biocompatibility of the C7 NPs (host) and Eu_10%_C7 NPs. These NPs were integrated into Hyalgan® to form H–C7 and H–Eu_10%_C7, collectively termed CartiOxgel. The formulated gel exhibited superior CT attenuation (∼1750–1900 Hounsfield units, HU), significantly outperforming Omnipaque™ (H-Iodine, ∼890 HU). *In vivo* X-ray CT imaging in Wistar rats following IA injection of CartiOxgel demonstrated sustained retention up to 48 h compared with the rapid clearance of H-Iodine. *Ex vivo* optical imaging revealed that the Eu_10%_C7 NPs exhibited effective tissue penetration due to the hypersensitive transition (^5^D_0_ → ^7^F_2_) of _10%_Eu. Histological analyses (H&E and Safranin-O staining) confirmed the absence of inflammatory response or extracellular matrix (ECM) degradation after 48 h, indicating excellent biocompatibility and cartilage penetration. CartiOxgel with hybrid-NPs holds promise for advanced IA imaging and has potential clinical applications in knee diagnostics.

## Abstract 2

CartiOxgel, a hybrid nano-contrast agent (EuC NPs in Hyalgan®), shows superior contrast and visibility in live X-ray CT imaging of Wistar rat knees post intra-articular injection, compared to commercial Omnipaque™ iodine.

## Graphical abstract

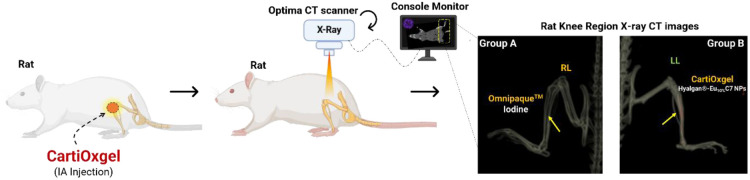

## 1. Introduction

Intra-articular (IA) imaging is essential for assessing joint health, diagnosing pathologies, and monitoring therapeutic interventions. However, the inherently low contrast between the synovial fluid, cartilage, and surrounding soft tissues poses significant challenges for conventional imaging modalities.^1,2^ This limitation hinders detailed visualization of IA structures, making accurate diagnosis and evaluation difficult. To address these challenges and enhance image contrast, exogenous contrast agents are introduced into the joint space. These agents improve differentiation within the IA space, facilitating better assessment of joint integrity and function. Currently, iodine-based contrast agents such as Omnipaque™, Ultravist®, Visipaque™, and Isovue® are widely used in clinical practice. However, they have several limitations, including rapid diffusion from the joint space, which limits the imaging window and potential adverse reactions.^3^ These drawbacks highlight the need for more effective and safer IA contrast agents.

Nanoprobes have emerged as powerful tools in biomedical imaging, offering enhanced diagnostic and therapeutic capabilities. In IA imaging applications, nanoparticles (NPs) serve as promising contrast agents due to their unique physicochemical properties, including high X-ray attenuation, tunable optical emission, and biocompatibility. Their nanoscale size enables better penetration into the articular cartilage, potentially facilitating early detection of joint disorders such as osteoarthritis (OA). However, the development of effective IA contrast agents remains a challenge due to retention, targeting, and imaging efficiency.^4^ Recent studies have demonstrated that nanoprobes outperform traditional iodine-based agents in contrast efficiency. For instance, polymer-coated ultrasmall cerium oxide (CeO_2_) NPs achieved an attenuation of 344 HU at 52.1 mM, compared to 487 HU for Ultravist® at 100 mM, highlighting the higher per-molecule efficiency of CeO_2_-based agents.^5^ However, CeO_2_ NP antioxidant properties and their long-term effects on joint health remain unclear. Tantalum oxide (Ta_2_O_5_ NPs),^6–8^ gadolinium oxide (Gd_2_O_3_),^9^ and terbium (Tb)^10^ offer advanced contrast properties for X-ray-based imaging, addressing the limitations of traditional contrast agents. Additionally, GdEu_*x*_Tb_1−*x*_O_3_ NPs demonstrated X-ray attenuation efficiencies of ∼10 HU mM^−1^, compared to 4.0–5.9 HU mM^−1^ for Ultravist®, further establishing their potential.^11^ However, while Eu-based fluorescence enables imaging in the visible range, its applicability for deep-tissue penetration has not been extensively explored. Moreover, single-mode imaging agents (*e.g.*, only X-ray or only fluorescence) are insufficient for comprehensive IA assessment. The combination of X-ray contrast (for deep penetration) and fluorescence imaging (for high-resolution surface mapping) provides superior diagnostic capabilities. To address the limitations of previous studies and overcome practical challenges in IA imaging, we have developed a multifunctional hybrid nanoprobe for dual imaging.

Rare-earth element-based bioinert nanomaterials have emerged as promising candidates for biomedical imaging. Cerium oxide NPs (CeO_2−*x*_ NPs) serve as an ideal host material due to their intrinsic redox activity (Ce^3+^/Ce^4+^),^12,13^ antioxidant properties,^14^ and biocompatibility, while europium (Eu) is widely recognized for its strong luminescent characteristics.^15,16^ The incorporation of Eu into CeO_2−*x*_ enhances photoluminescence,^17^ antioxidant activity, and X-ray attenuation, making europium-doped cerium oxide nanoparticles (Eu_*x*%_C NPs) promising candidates for multimodal imaging applications. Despite the availability of various synthetic strategies, including plasma-electrochemical synthesis,^18^ hydrothermal methods,^19^ chemical precipitation,^20^ and the oxalate precursor method,^21^ achieving scalable production with uniform Eu doping in CeO_2−*x*_ remains a challenge. To the best of our knowledge, combustion-based Eu doping into CeO_2−*x*_ NPs has not been previously explored. In this study, we employed a one-pot combustion method to enhance scalability, reproducibility, and structural stability. The synthesis was followed by thermal treatment to refine the crystallinity and optimize the physicochemical properties. The resulting Eu_*x*%_C NPs exhibited a distinct bounded morphology with an average particle size of ∼20 nm, facilitating efficient penetration across the layers of the cartilage tissue. A comprehensive characterization of the synthesized Eu_*x*%_C NPs was performed to evaluate the influence of dopant concentration and thermal treatment on their X-ray and optical performance.

While metal oxide NPs typically exhibit poor affinity for cartilage extracellular matrix (ECM) components such as collagen, proteoglycan, or glycosaminoglycans (GAGs), they are distributed unevenly without targeted modifications, thereby reducing imaging accuracy.^22^ We address this limitation by incorporating NPs into Hyalgan®, a commercially available, FDA-approved IA gel composed of sodium hyaluronate. Hyalgan® exhibits shear-thinning behavior, high viscosity (50–100 cP at 25 °C), and an optimal molecular weight (500–730 kDa), facilitating its diffusion into cartilage and enhancing lubrication, hydration, and mechanical support.^23,24^ The integration of Eu_*x*%_C NPs into Hyalgan® results in a hybrid NP gel formulation, termed CartiOxgel. CartiOxgel was characterized to evaluate its impregnation efficiency and X-ray attenuation properties. It was administered as an IA injection in healthy adult rats and compared with the clinically used iodine-based contrast agent, Omnipaque™. CartiOxgel demonstrated superior X-ray CT attenuation and optical properties, with sustained retention up to 48 h post-injection. Histological analysis confirmed internalization of NPs within the cartilage, and their biological effects were quantitatively assessed through histopathological scoring. This study presents the first reported investigation of CartiOxgel as a dual-mode contrast agent, demonstrating its potential for IA applications in X-ray and optical imaging in animal models.

## 2. Experimental method

### 2.1 Materials

All reagents used in this study were of analytical grade and were used without further purification. Cerium(iii) nitrate hexahydrate (Ce (NO_3_)_3_·6H_2_O), europium(iii) nitrate hexahydrate (Eu (NO_3_)_3_·6H_2_O), and silicone oil (330–370 cps) were procured from Sisco Research Laboratories. Diethylene glycol (DEG, C_8_H_18_O_3_) was obtained from Fisher Scientific, and nitric acid (HNO_3_, 99% purity) and citric acid monohydrate (C_6_H_8_O_7_·H_2_O) were obtained from Merck. Deionized water was used as the solvent medium, and phosphate-buffered saline (PBS) was prepared as needed. For cell culture experiments, Dulbecco's Phosphate Buffered Saline (DPBS), Dulbecco's Modified Eagle Medium (DMEM), Fetal bovine serum (FBS), penicillin-streptomycin, and Thiazolyl Blue Tetrazolium Bromide (MTT) were obtained from Sigma-Aldrich. Sterile 31-gauge needles and 40-U syringes (Dispo Van®) were used for intra-articular (IA) injections in animal studies. Omnipaque™ (350 mg I per mL), an iodine-based contrast agent, was purchased from GE Healthcare. Hyalgan®, a Sodium Hyaluronate E.P. (20 mg/2 mL) with a molecular weight of 500–730 kDa, was also used. All chemicals were used in accordance with the specific requirements of the experimental protocols.

### 2.2 Animal experiments and ethics

Female Wistar rats (12 weeks old, weighing 150–200 g) were procured from Mass Biotech, Tamil Nadu, India (Reg No. 2084/PO/RcBt/S/19/CPCSEA). All animal experiments were approved by the Institutional Animal Ethics Committee (IAEC) of SRM College of Engineering (Approval No. SAF/IAEC/240124/011). Rats were housed in a specific pathogen-free (SPF) facility under controlled conditions (23 °C ± 3 °C, 12-hour light/dark cycle with standard food and water). These standardized conditions ensured the well-being of the animals and the integrity of the experimental data.

### 2.3 Synthesis of cerium oxide nanoparticles (CeO_2−*x*_ NPs, C)

CeO_2−*x*_ NPs were synthesized *via* a solvent-assisted combustion process, as described in our previous publication.^25^ Ce (NO_3_)_3_·6H_2_O and citric acid monohydrate were dissolved in HNO_3_, followed by DEG addition. The mixture was heated at 200 °C in a silicon oil bath, initiating self-ignition and forming the as-prepared powder (C2 NPs). Subsequently, the C2 NP sample was annealed at 700 °C for 30 min to obtain the thermally treated product (C7 NPs). The selection of 700 °C as the thermal treatment temperature was based on prior studies with undoped CeO_2−*x*_ NPs demonstrating enhanced crystallinity and bioimaging performance.

### 2.4 Synthesis of europium-doped cerium oxide nanoparticles (Eu_*x*%_Cy NPs)

Europium-doped CeO_2−*x*_ NPs (Eu_*x*%_Cy NPs) were synthesized using the same combustion technique. Eu (2% and 10%) was added at specific weight proportions relative to the cerium precursor. To achieve 2% doping, 727.6 mg of Ce (NO_3_)_3_·6H_2_O was combined with 14.553 mg of Eu (NO_3_)_3_·6H_2_O. To achieve 10% doping, 727.6 mg of cerium nitrate was mixed with 72.76 mg of europium nitrate. Each precursor mixture was dissolved in 10 mL of 0.04 M HNO_3_ separately and stirred continuously. Subsequently, 354.0 mg of citric acid monohydrate was added to create a homogenous solution. Then, 0.02 mL of DEG was added dropwise to control combustion kinetics and promote uniform nucleation. The resulting homogeneous solution was heated in a silicon oil bath at 200 °C for 2 h, triggering spontaneous self-ignition and yielding dusky yellow powders, which are referred to as Eu_2%_C2 NPs and Eu_10%_C2 NPs, respectively. The as-prepared powders were subjected to additional thermal treatment at 700 °C for 30 minutes, resulting in the production of pale-yellow powders referred to as Eu_2%_C7 NPs and Eu_10%_C7 NPs, respectively.

### 2.5 Characterization

#### 2.5.1 X-ray diffraction (XRD)

Cu Kα radiation was used to obtain powder XRD data using a BRUKER USA D8 Advance, Davinci. Peaks were produced within the 20°–90° range using a scanning rate of 0.05° s^−1^ and a wavelength of 1.5406 Å. The sample peaks were analyzed using X'Pert High Score software. The Full Width at Half Maximum (FWHM) values were used to calculate the lattice parameters, determine the crystallite size using the Scherrer equation, and estimate the microstrain through Williamson–Hall analysis. The sample peak positions (C2, C7, Eu_2%_C2, Eu_2%_C7, Eu_10%_C2 and Eu_10%_C7 NPs) were cross-referenced with the CeO_2_ NPs from the JCPDS reference under entry number 34-0394.

#### 2.5.2 High-resolution transmission electron microscope (HR-TEM)

An HR-TEM (JEM-JEOL-2100 Plus, Japan) coupled with an energy-dispersive spectrometer (EDS) was used to analyze the morphology of C7 and Eu_10%_C7 NPs. The nanoparticles were uniformly suspended by dispersing them in ethanol and sonicating them for five minutes. The resulting dispersion was deposited on a carbon-coated copper grid and allowed to dry completely at room temperature before imaging. ImageJ software was used to determine the particle size distribution. Furthermore, a scanning transmission electron microscopy (STEM) analysis was performed on Eu_10%_C7 NPs to verify doping through elemental mapping.

#### 2.5.3 Zeta potential and dynamic light scattering (DLS)

The zeta potential and DLS of C2, C7, Eu_2%_C7, and Eu_10%_C7 NPs dispersed in a 1 : 1 (v/v) water–ethanol mixture was measured using a Malvern Zeta sizer Nano ZS-90. This solvent combination was selected to improve polarity and reduce NP aggregation by balancing dispersion stability and reducing surface tension. Each sample (1 mg mL^−1^) was ultrasonicated for 30 minutes before measurement to ensure maximum dispersion. DLS was performed at 25 °C with a scattering angle of 90° to determine the hydrodynamic diameter and polydispersity index (PDI). The zeta potential was determined through electrophoretic mobility using a disposable zeta cell cuvette at the same temperature. Despite ultrasonication and solvent optimization, considerable clustering remained due to the intense surface reactivity of the NPs.

#### 2.5.4 X-ray photoelectron spectroscopy (XPS)

The PHI Versa Probe III system (Japan) was employed to conduct X-ray photoelectron spectroscopy (XPS) measurements. This system was equipped with a monochromatic Al Kα X-ray source (1486.6 eV) in a dual-anode configuration. To determine the dopant and atomic ratios of the as-prepared and annealed C2, C7, Eu_2%_C7, and Eu_10%_C7 NPs. Survey spectra were obtained at a pass energy of 100 eV to determine the elemental composition, and high-resolution scans were performed at a pass energy of 55 eV to resolve the Ce 3d, Eu 4d, and O 1s core levels. Data analysis was conducted using OriginPro, including peak fitting, oxidation state evaluation, and ratio calculation from integrated peak areas using1
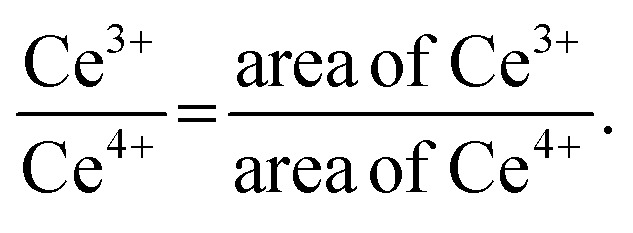


#### 2.5.5 Fourier transform infrared spectrometer (FTIR)

FTIR analysis was performed using a Shimadzu IRTracer-100 to examine the molecular interactions and bonding characteristics of the samples. The analysis included C7 NPs, Eu_10%_C7 NPs, Hyalgan®, H-Iodine, H–C7, and H–Eu_10%_C7 to assess the impregnation of metal oxide within the Hyalgan® matrix. Transmittance spectra were recorded over the mid-infrared range of 400–4500 cm^−1^ to identify functional groups and evaluate chemical bonding variations. The spectral data were analyzed and plotted using OriginPro software to investigate the impact of impregnation of the doped and undoped CeO_2−*x*_ NPs.

### 2.6 Spectroscopic analysis

#### 2.6.1 UV-visible-NIR spectroscopy

A PerkinElmer LAMBDA 950 spectrometer was used to record the UV-visible-NIR absorbance spectra of C2, C7, Eu_2%_C7, and Eu_10%_C7 NPs in the 200–1000 nm range. A solution was prepared for each sample by sonicating 1 mg of nanopowder with 3 mL of deionized water.

#### 2.6.2 Photoluminescence spectroscopy (PL)

The PL emission spectra of C2, C7, Eu_2%_C7, and Eu_10%_C7 NPs were recorded using a Jobin Yvon Fluorimeter (France) with an excitation wavelength of 466 nm and an emission range of 400–700 nm. For analysis, 1 mg of each nanopowder was ultrasonicated in 3 mL of deionized water to ensure uniform dispersion.

#### 2.6.3 Photoluminescence (PL) lifetime measurements

Time-resolved PL lifetime measurements using a Jobin Yvon Fluorimeter (France) were performed for C2, C7, Eu_2%_C7, and Eu_10%_C7 NPs. Using a decay fitting model 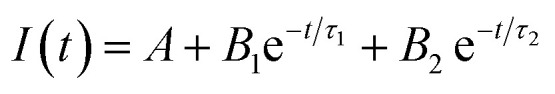, where *τ*_1_ and *τ*_2_ represent the lifetimes of different emission states. The measurements were conducted with a time calibration of 0.049 ns per channel and within a dynamic range of 0–1023 channels. The data fitting range was adjusted for each sample to exclude noise and improve accuracy. Key parameters, including peak count, total count, and experimental time, were recorded for each sample. The analysis provided a comprehensive understanding of the emission dynamics and quenching behavior in undoped (C2, C7 NPs) and doped (Eu_2%_C7, Eu_10%_C7 NPs).

### 2.7 Evaluation of antioxidant activity using DPPH assay

The antioxidant activity of C2, C7, Eu_2%_C7, and Eu_10%_C7 NPs was evaluated using the 2,2-diphenyl-1-picrylhydrazyl (DPPH) assay, which was adapted and modified from the literature.^26^ A 0.1 mM DPPH stock solution was prepared in ethanol and adjusted to an initial absorbance of approximately 0.973 at 517 nm. NP suspensions were prepared in a 1 : 1 (v/v) water–ethanol mixture at concentrations of 5.0, 2.5, 1.25, 0.625, and 0.3125 mg mL^−1^. From each concentration, 0.1 mL of the respective NP suspension was added to individual wells of a 96-well microplate, followed by 0.02 mL of the DPPH solution. The plate was incubated in the dark at room temperature for 30 minutes to minimize photodegradation of the DPPH radical. After incubation, absorbance at 517 nm was measured for each well using a Thermo Scientific™ Multiskan™ GO microplate spectrophotometer (Fig. S7A–C†).2
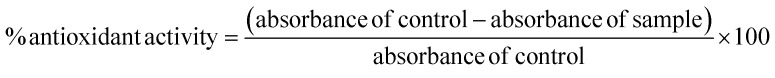


### 2.8 Preparation of intra-articular (IA) injection

Commercially available Hyalgan® was used as the base to formulate IA injectable hydrogels. The NPs-incorporated hydrogel formulations were referred to as “CartiOxgel”. For preparation, four vials of Hyalgan® (10 mg mL^−1^) were pooled to obtain sufficient volume and ensure uniform formulation consistency. For the H-Iodine formulation, 0.057 mL of Omnipaque™ iodine(i) (350 mg I per mL) was diluted with Hyalgan® to yield a final iodine concentration of 20 mg mL^−1^ in the resulting mixture. For the CartiOxgel formulations, 20 mg of C7 NPs or Eu_10%_C7 NPs were directly incorporated into Hyalgan®. CartiOxgel (20 mg mL^−1^) was vortexed for 5 minutes to ensure homogeneous dispersion of nanoparticles and removal of air bubbles. IA injection formulations were prepared under aseptic conditions and stored at 4 °C until use in preclinical imaging studies. Each formulation was loaded into a sterile syringe fitted with a 31 G needle, and 0.05 mL was administered intra-articularly, delivering 1 mg/0.05 mL of the respective formulation per injection.

### 2.9 Evaluation of the attenuation rate using CT

To evaluate the X-ray attenuation properties of the synthesized nanoprobes, powdered samples of C2, C7, Eu_2%_C7, and Eu_10%_C7 NPs were accurately weighed (5 mg each) and placed into individual 0.2 mL microcentrifuge tubes. For comparative analysis, Omnipaque™ iodine-based clinical contrast agent (350 mg I per mL)—was diluted to deliver an equivalent iodine content of 5 mg. Distilled water was used as a negative control. Computed tomography (CT) scans were performed using a 64-slice GE Bright Speed Optima CT660 CT scanner. The imaging parameters were set at a tube Voltage of 80 kV, a tube current of 95 mA, and a slice thickness of 0.6 mm with a region of interest (ROI) of 0.104 cm^2^. Similarly, the CartiOxgel samples: Hyalgan®, H-Iodine, H–C7, and H–Eu_10%_C7 were examined. All samples were scanned under identical conditions.

#### 2.9.1 Image analysis

The CT images were analyzed using the 64-bit software version 16HW45.1 provided with the GE Optima CT660 CT scanner. For each sample, the Hounsfield Unit (HU) values were recorded from three distinct slices of the CT image. The average HU value for each sample was calculated based on these measurements. The CT attenuation values are reported as the average HU for each sample.

### 2.10 *In vitro* cell viability

NIH 3T3 mouse embryonic fibroblast cell lines were obtained from ATCC® and cultured in DMEM supplemented with 10% FBS and 1% penicillin–streptomycin. Cells were maintained under standard conditions in a humidified incubator at 37 °C with 5% CO_2_. The cells were washed with DPBS before the experiment to ensure sterility and remove the residual medium. To promote cell adhesion, fibroblast cells were seeded into 96-well plates at a density of 5000 cells per well and incubated under standard culture conditions for 24 h. Treatment solutions of C7 NPs, Eu_10%_C7 NPs, and CartiOxgel (Hyalgan®, H-Iodine, H–C7, and H–Eu_10%_C7) were prepared at concentrations of 0.02, 0.04, 0.08, 0.16, 0.32, 0.64, and 1.28 mg mL^−1^. Stock solutions of the NPs were initially prepared by suspending each sample in DPBS to a final concentration of 2.56 mg mL^−1^. The stock solutions were subjected to 30 seconds of sonication followed by three cycles of icing and re-sonication to ensure complete dispersion and homogeneity. Subsequent serial dilutions were performed in DMEM to achieve the required working concentrations. The cytotoxicity of the NP treatments was evaluated at 24 and 48 h intervals using the MTT assay. Following the incubation with treatment solutions, the MTT reagent (0.5 mg mL^−1^) was added to each well with the standard assay protocol. After the prescribed incubation period, the plates were centrifuged at 400 rpm for 2 minutes to ensure the removal of residual media. Post-centrifugation, DMSO was added to each well, mixed thoroughly and 50 μL of supernatant from each well was carefully transferred to new wells for spectrophotometric absorbance measurements. Absorbance was recorded at 570 nm using a microplate reader, with background correction applied based on wells containing media and treatment solutions in the absence of cells. All experiments were performed in triplicate, with three wells per treatment concentration containing cells, alongside three background control wells lacking cells. The data obtained from the assay were analyzed to assess the cytotoxic effects of the NPs on fibroblast cells, enabling a comparative evaluation of the nano-contrast agent.

### 2.11 CT imaging of the rat knee region

*In vivo* imaging experiments were performed using a 64-slice GE Optima CT660 CT scanner to assess the imaging efficacy of intra-articular (IA) administered CartiOxgel. Imaging was conducted on female Wistar rats (two groups: A and B, *n* = 3 per group, ∼160 g) at 0-, 24-, and 48-h post-injection. As previously described, the prepared IA injection was administered at a dose of 1 mg/0.05 mL per rat of H-Iodine, H–C7, or H–Eu_10%_C7. The CT scanning parameters consisted of 80 kV to 120 mA, 20.11 s exposure time, 0.62 mm collimation width, 20.0 mm total collimation width, and a pitch factor of 0.53. The scanned length and reconstructable Volume ranged from the top Z-locations of 110.64 and 102.31 mm to the bottom Z-locations of −99.04 mm and −90.81 mm, respectively. The CT images were analyzed using AW Volume Share 7 software (GE Healthcare), utilizing advanced 3D hardware and volume rendered (VR) configurations to assess the localization and retention of the contrast agents at the knee joint. The HU values were analyzed and plotted using the ROI analysis to evaluate the distribution and retention of the agents over time post-injection.

### 2.12 *Ex vivo* optical imaging

Following euthanasia, the knee regions of the animals were excised for *ex vivo* analysis. Optical imaging was performed using the *in vivo* Imaging System (IVIS) to evaluate the fluorescence properties of the IA-injected CartiOxgel at 48 h post-injection. The system was configured with an excitation wavelength of 466 nm and an emission filter set to DsRed. Epi-illumination was used with the lamp level set too high. Imaging parameters included a binning level of 8 (HS), field of view (FOV) of 12.5 cm, f-stop of 2, and exposure time of 15 seconds. The standardized settings ensured the consistency and repeatability of the fluorescence data. Quantitative analysis was performed using IVIS Living Image® software.

### 2.13 Histological examination

Following optical imaging of the rat knee region, the tissues surrounding the knee joint were dissected to isolate the articular cartilage for evaluating the internal effects of IA-administered CartiOxgel. The cartilage was fixed in 4% formalin for 4 h and decalcified in 10% nitric acid for 24 h. After thorough rinsing, the samples were embedded in paraffin wax, stored at −80 °C, and sectioned into 1 μm slices using a microtome. Safranin O staining was performed to assess glycosaminoglycan (GAG) content, while hematoxylin and eosin (H&E) staining was used to evaluate structural integrity. The stained sections were imaged using a phase-contrast microscope to examine the cartilage morphology and GAG distribution. The quantification and assessment of internalization were conducted using the Mankin scoring method under the supervision of a pathologist.

### 2.14 Statistical analysis

Statistical analysis was conducted using GraphPad Prism Software, version 8.02 (San Diego, CA, USA). Data are expressed as mean ± standard deviation (SD). Differences in three groups (*n* = 3) were analyzed using one-way analysis of variance (ANOVA) followed by post hoc comparisons. A *p*-value of less than 0.05 was considered statistically significant.

## 3. Results and discussion

### 3.1 Synthesis of Eu_*x*%_Cy NPs

**Fig. 1:**
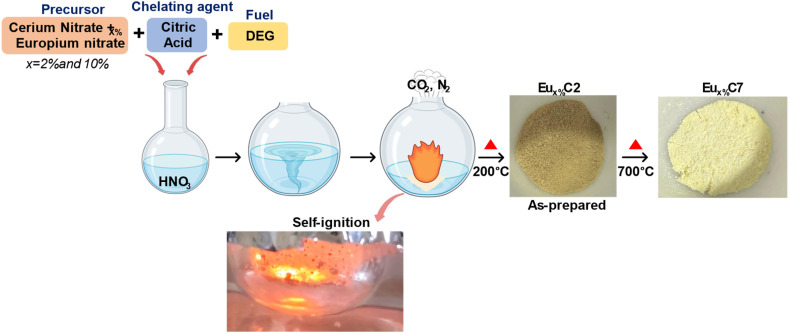
Diagrammatic representation of the one-pot solution combustion synthesis method with thermal treatment. The resulting nanopowders are labelled as Eu_*x*%_C2 NPs (dusky yellow) and Eu_*x*%_C7 NPs (pale yellow) post-thermal treatments at 200 °C and 700 °C, respectively.

The synthesis of Eu_*x*%_Cy NPs using the one-pot solution combustion method is illustrated in Fig. 1. This method follows a redox-based exothermic reaction in which metal salts, oxidizers, and fuel interact to facilitate NP formulation. Cerium and Europium nitrate dissolved in HNO_3,_ where NO_3_^−^ acts as an oxidizer, facilitating the incorporation of Ce and Eu ions in the solution. DEG acts as a coordinating solvent and secondary fuel, playing a critical role in moderating the combustion rate.^27^ Initially, as the mixture was heated to ∼150 °C, water and HNO_3_ evaporated, facilitating the proper metal ion reduction and maintaining the Ce^3+^/Ce^4+^ ratio in the host. Citric acid functions as a complexing and reducing agent. Ion reduction occurs through electron donation from citric acid, enhancing oxygen vacancy (*V*_o_) formation.^28^ The presence of *V*_o_ enables Eu^3+^ (atomic radius = 1.07 Å) substitution at Ce^4+^ (atomic radius = 0.97 Å) sites, to stabilize the host structure.^29^ Upon further heating, citric acid and DEG decompose, and at approximately 185 °C-200 °C, self-ignition occurs with an orange smoldering flame and rapid gas evolution. The combustion of carbon-based fuels by nitrate-derived oxygen releases CO_2_, whereas N_2_ is formed *via* thermal decomposition and secondary reduction of NO_3_^−^ and NO_*x*_ species. This exothermic reaction rapidly converts the liquid precursor into a fine, dusky yellow powder (Eu_2%_C2 and Eu_10%_C2 NPs). Post-annealing at 700 °C (Eu_2%_C7 and Eu_10%_C7 NPs) improves crystallinity, removes residual carbon, and yields pale-yellow nanocrystalline powders.

### 3.2 Characterization

#### 3.2.1 Structural analysis

**Fig. 2:**
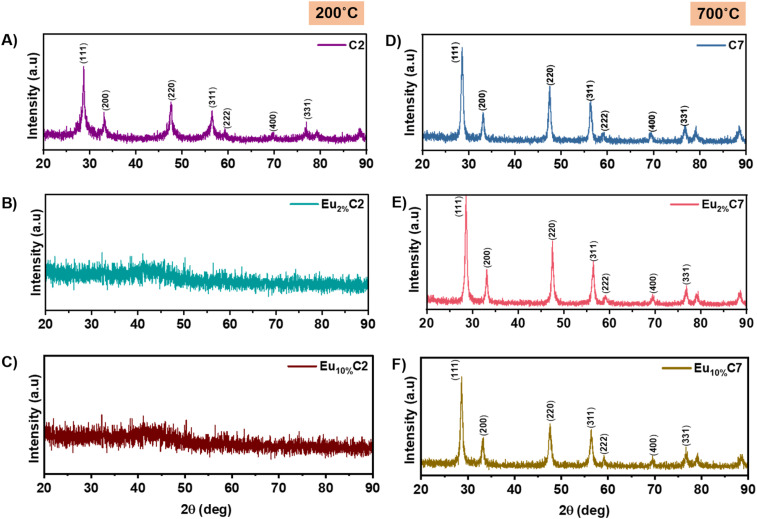
XRD patterns of undoped CeO_2−*x*_ NPs (C2 NPs, C7 NPs) and Eu-doped CeO_2−*x*_ NPs (2% and 10%) after thermal treatments at 200 °C and 700 °C. (A) C2 and (D) C7 represent undoped samples; (B and C) Eu_2%_C2 and Eu_10%_C2; (E and F) Eu_2%_C7 and Eu_10%_C7 correspond to the Eu-doped samples.

The XRD patterns of the Eu_*x*%_Cy NPs with low thermal treatment (C2, Eu_2%_C2, Eu_10%_C7 NPs) and high thermal treatment (C7, Eu_2%_C7, Eu_10%_C7 NPs) are shown in Fig. 2. Based on the JCPDS reference pattern 34-0394,^30^ we indexed all of the reflections to ceria's cubic fluorite phase (*Fm*3*m* space group). The diffraction peaks at 2*θ* values of 28.5°, 33.12°, 47.4°, 56.34°, 59.11°, 69.4°, 76.8°, 79.12°, and 88.6° correspond to the (111), (200), (220), (311), (222), (400), and (311) lattice planes, respectively. No europium oxide or hydroxide peaks were detected, confirming the CeO_2−*x*_ lattice stability upon Eu doping. However, a subtle variation in the 2*θ* peak of the (111) lattice plane was observed. In undoped C2 NPs, the peak appeared at 28.74° and shifted to 28.59° in C7 NPs. For Eu_2%_C7 NPs, the peak shifted to 28.50°, indicating lattice expansion due to Eu^3+^ substitution. At higher doping (Eu_10%_C7 NPs), the shift to 28.60° was due to strain relaxation.

The C2 NPs showed broader and sharper peak patterns (Fig. 2A), whereas the Eu_2%_C2 NPs and Eu_10%_C2 NPs exhibited an amorphous pattern due to the hindered crystallization caused by insufficient atomic mobility (Fig. 2B and C). Limited thermal activation slows nucleation, preventing lattice formation and leaving the material in a disordered, high-energy amorphous state.^31^ NP heat treatment at 700 °C (C7, Eu_2%_C7, and Eu_10%_C7 NPs) resulted in narrow and sharp crystalline peaks (Fig. 2D–F). The increased thermal energy promotes structural relaxation,^32^ where Eu^3+^ doping creates pinning centers within the lattice. Eu^3+^ substitution induces *V*_o_, prompting atomic rearrangement into an ordered structure to maintain charge neutrality and minimize lattice strain.^33^ Unlike Ostwald ripening, this interaction stabilizes the crystal without significant grain growth. The lattice parameter remained constant at ∼5.40 Å (Fig. S1B†), indicating a balance between Eu^3+^-induced expansion and the compensatory effect of *V*_o_, which enhances structural stability.

The crystallite size, calculated using the Scherrer equation (Fig. S1B†), decreased from 27.73 nm (C2 NPs) to 20.20 nm (C7 NPs), with further reduction in doped samples, 21.33 nm for Eu_2%_C7 NPs and 17.44 nm for Eu_10%_C7 NPs. This trend indicates that Eu^3+^ doping and thermal treatment suppress grain growth by introducing pinning centers (Eu^3+^ and *V*_o_), thereby inhibiting particle coalescence during crystallization.^34^ Conventional methods generally yield larger crystallites due to slower reaction kinetics and prolonged growth durations. The microstrain increased from 3.5 × 10^−6^ (C2 NPs) to 4.16 × 10^−6^ (C7 NPs) and further to 3.85 × 10^−6^ (Eu_2%_C7 NPs) and 4.76 × 10^−6^ (Eu_10%_C7 NPs), reflecting the higher defect densities and localized lattice distortions induced by the rapid combustion synthesis. The inverse correlation between crystallite size and microstrain suggests that increased surface stress and grain boundary defects influence the structural adaptability of the fluorite lattice.

#### 3.2.2 Morphological analysis

**Fig. 3:**
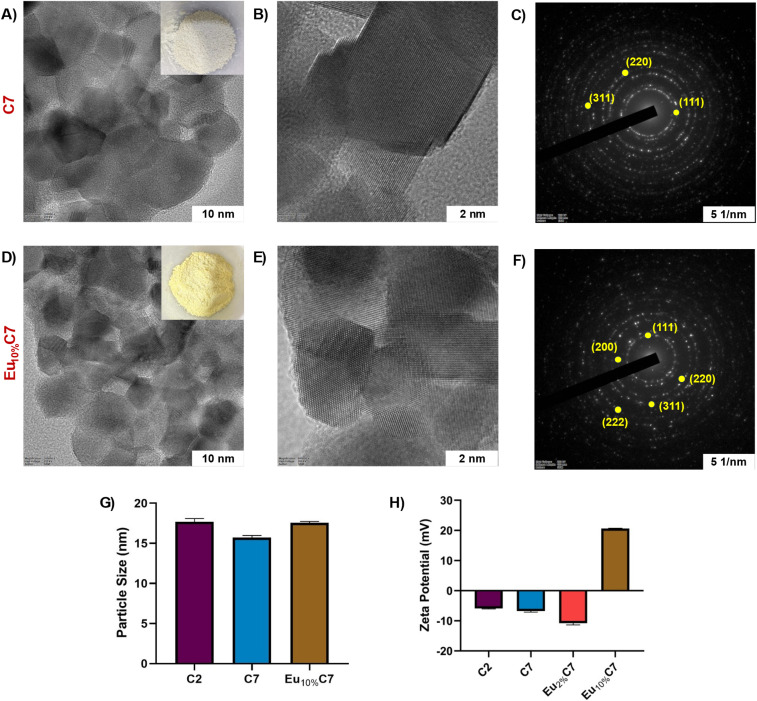
Morphological analysis using HR-TEM: (A and B) HR-TEM images of host C7 NPs, with the inset in A showing the powdered sample. Scale bars = 10 nm and 2 nm. (C) SAED pattern showing lattice planes, scale bar = 5 1/nm. (D and E) HR-TEM images of Eu_10%_C7 NPs, with the inset in B showing the powdered sample. Scale bars = 10 nm and 2 nm. (F) SAED pattern showing lattice planes, scale bar = 5 1/nm. (G) Particle size distribution of host C2, thermally treated C7 and doped Eu_10%_C7 NPs. (H) Zeta potential of undoped (C2 and C7 NPs) and doped (Eu_2%_C7 and Eu_10%_C7 NPs).

The morphology of undoped and doped NPs was analyzed using HR-TEM and is shown in Fig. 3. The size and morphology of undoped and doped NPs are regulated by the fuel-oxidizer ratio and DEG, which control the nucleation kinetics in one-pot synthesis. As reported in our previous publication, C2 NPs exhibited larger, polyhedral-shaped agglomerates with an average particle size of 17.74 ± 3.07 nm.^25^ Thermal treatment of C7 NPs (15.40 ± 2.3 nm) and Eu_10%_C7 NPs (17.67 ± 1.06 nm) resulted in polyhedral-shaped, uniform particles with refined particle boundaries (Fig. 3G). The observed agglomeration in Fig. 3A and D was primarily due to the increased electron density at the NP surface, a result of *V*_o_ defects. The addition of surfactants reduces agglomeration, alters the surface chemistry and potentially impacts the intrinsic properties of the NPs. Therefore, to better understand the interfacial characteristics, Fast Fourier Transform (FFT) analysis of HR-TEM images was performed (Fig. S2†). C7 NPs displayed well-defined lattice fringes (Fig. S2B†), whereas Eu_10%_C7 NPs showed slightly expanded fringes (Fig. S2D†). The measured interplanar spacing increased from 0.356 nm (C7 NPs) to 0.417 nm (Eu_10%_C7 NPs), indicating lattice distortion or interfacial strain. This expansion was attributed to the substitution of Ce^4+^ with larger Eu^3+^ ions, confirming the incorporation of effective dopants, particularly at particle surfaces and interfaces. The selected area electron diffraction (SAED) patterns (Fig. 3C and F) confirm the fluorite cubic structure, with diffraction rings corresponding to the (111), (200), (220), (311), and (222) planes, indicating high crystallinity without any structural distortions from doping. STEM analysis and elemental mapping (Fig. S3†) demonstrate uniform Eu distribution within the CeO_2−*x*_ matrix, while energy-dispersive X-ray (EDX) analysis (Fig. S4†) confirmed Eu incorporation. The decreased oxygen content in Eu_10%_C7 NPs (Fig. S4B†) indicates the formation of *V*_o_ to compensate for Eu^3+^ substitution. The synthesized NPs are less than 20 nm, which is in the optimal size range for penetrating the superficial zone of articular cartilage (50–60 nm) and the proteoglycan network (∼20 nm).^35^ Thus, the developed NPs facilitate effective transport and interaction within the cartilage matrix.

#### 3.2.3 Stability evaluation

Table S1† summarizes the hydrodynamic diameter (*d*, nm), polydispersity index (PDI), and zeta potential values. C2 NPs exhibited the lowest stability with a large hydrodynamic size of 1264 ± 1126 nm and a high PDI of 0.58, indicating significant polydispersity. Weak electrostatic repulsion (−6.02 mV) increases the tendency for aggregation and reduces the stability of long-term dispersion. Thermally treated C7 NPs reduce surface defects, resulting in a narrower size distribution (925.9 ± 168.3 nm, d nm) with a slightly reduced PDI of 0.519. However, with a zeta potential of −6.51 mV, the electrostatic repulsion remains insufficient for prolonged stability. In contrast, Eu_2%_C7 NPs showed improved stability with a hydrodynamic size of 1044 ± 136.1 nm and a low PDI of 0.124 with monodispersity. A zeta potential of −11.8 mV strengthened the electrostatic repulsion and minimized the aggregation. Among all the samples, Eu_10%_C7 NPs exhibited the highest stability, with a zeta potential of +20.7 mV (Fig. 3H). Despite a moderate PDI of 0.274 and a hydrodynamic size of 1186 ± 361.3 nm, the strong positive surface charge provided significant electrostatic repulsion, preventing aggregation and ensuring dispersion stability.

#### 3.2.4 Surface charge dynamics and effect of Eu doping

The negative zeta potentials of C2, C7, and Eu_2%_C7 NPs are attributed to surface Ce^3+^ ions and *V*_o_. These *V*_o_ tend to attract hydroxyl groups (OH^−^) in aqueous or ethanol media. The addition of 2% Eu slightly increases the negative surface charge by modulating the defect states, raising the zeta potential to −11.8 mV. The substantial shift to +20.7 mV in Eu_10%_C7 NPs can be attributed to the increased incorporation of Eu^3+^ ions into the host lattice. Eu^3+^ is highly electropositive compared to cerium, and thus, increased Eu (10%) content reduces surface *V*_o_ and alters charge distribution, which can lead to a net positive surface potential, thereby preventing aggregation and significantly improving colloidal dispersion stability. Thus, the charge and dispersion stability of Eu-doped NPs make them suitable for impregnation into the IA gel and reflect the potential for stable retention in the synovial fluid environment.

### 3.3 XPS analysis

The oxidation state and stabilization effects of 2% and 10% Eu doping in combustion-synthesized Eu_*x*%_Cy NPs were investigated using XPS. The XPS survey spectra (Fig. S6†) confirmed the presence of Ce, Eu, and O atoms. Deconvolution of the Ce 3d and Eu 3d spectra revealed six peaks (*u*_3_, *u*_2_, *u*_1_, *v*_3_, *v*_2_, *v*_1_) and four peaks (*w*_2_, *w*_1_, *z*_2_, *z*_1_), respectively, with binding energies and integrated areas (Table S2†).

#### 3.3.1 Ce 3d spectra analysis

**Fig. 4:**
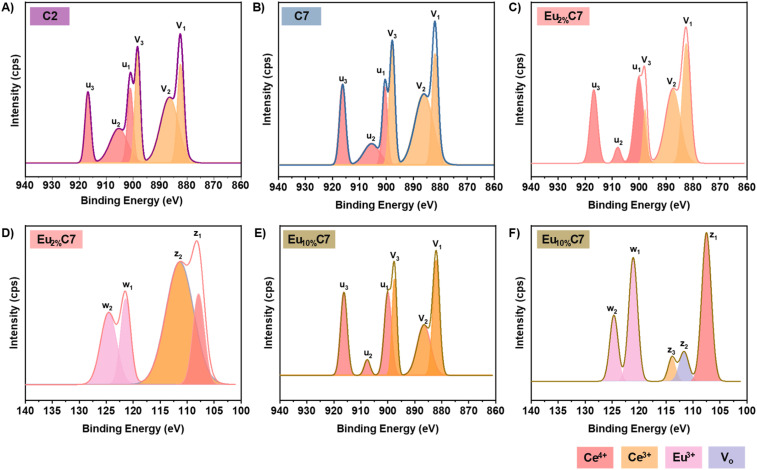
XPS spectra of host the C2 and C7 NPs (A and B) and the doped Eu_*x*%_Cy NPs (Eu_2%_C7 and Eu_10%_C7 NPs) (C–F). (A and B) Ce 3d region of the C2 and C7 NPs. (C and E) Ce 3d region of the Eu_2%_C7 and Eu_10%_C7 NPs. (D and F) Eu 4d spectra of the Eu_2%_C7 and Eu_10%_C7 NPs. The color scheme for all fitted curves is provided for clarity.

The Ce 3d spectra exhibited characteristic peaks corresponding to Ce^3+^ and Ce^4+^ states, confirming the mixed-valence nature of CeO_2−*x*_ NPs (Fig. 4). Notable changes were observed between C2 and C7 NPs, indicating the influence of annealing on the electronic structure and surface chemistry.^36^ In C7 NPs, the *u*_3_ peak remained sharp, indicating the retention of the Ce^4+^ lattice structure (Fig. 4B). However, the reduced intensities of the *u*_2_ and *u*_1_ peaks suggest a decrease in the surface Ce^4+^ state. Conversely, the *v*_3_, *v*_2_, and *v*_1_ peaks showed a slight increase, indicating an enhancement in Ce^3+^ sites due to increased *V*_o_ following thermal treatment, which stabilized the Ce^3+^ state. The Ce^3+^/Ce^4+^ ratio decreased from 2.08 in C2 to 1.62 in C7 NPs, suggesting a relative increase in Ce^4+^ content due to reduced *V*_o_ following high-temperature treatment (Fig. 4A). The O 1s spectra showed a broad peak at ∼529 eV in C2 NPs, attributed to defect-related oxygen species (Fig. S4B†). In C7 NPs, the peak at ∼529 eV became sharper and shifted to lower binding energy after thermal treatment, consistent with a reduction in *V*_o_ (Fig. S4D†).

#### 3.3.2 Effect of Eu doping in Ce 3d spectra

The incorporation of Eu dopants altered the Ce 3d spectra of Eu_2%_C7 NPs (Fig. 4C) and Eu_10%_C7 NPs (Fig. 4E). Increasing Eu doping from 2% to 10% led to decreased Ce^3+^ peak intensities and stabilized the Ce^4+^ state. The emergence of *u*_2_ as a distinct peak in Eu_2%_C7 NPs and Eu_10%_C7 NPs is attributed to Eu interaction with the electronic state of the host lattice. In addition, reduced *u*_1_ and increased *v*_2_ peak intensities in Eu_10%_C7 NPs indicate lattice distortion and enhanced Ce^4+^ stabilization.^37^ The O 1s spectra showed lattice oxygen at ∼529–530 eV and *V*_o_ at 531–532 eV (Fig. S4F†). The *V*_o_ peak was broad in C2, reduced in C7, and pronounced in Eu_2%_C7 NPs due to lower doping, whereas no visible *V*_o_ peak was observed in Eu_10%_C7 NPs (Fig. S4H†). The integrated area analysis revealed a clear shift towards higher Ce^4+^ dominance with increasing Eu doping.^16^ The Ce^3+^/Ce^4+^ ratio decreased from 1.25 in Eu_2%_C7 NPs to 0.85 in Eu_10%_C7 NPs, indicating that higher Eu^3+^ incorporation promoted Ce^4+^ stabilization by reducing *V*_o_.

#### 3.3.3 Eu 4d spectra analysis

The Eu 4d fitted spectra provide critical insights into the incorporation of Eu ions within the ceria lattice. Fig. 4D shows that the Eu_2%_C7 NPs exhibit doublet peaks at 124.54 eV (*w*_2_) and 121.37 eV (*w*_1_), corresponding to the Eu 4d_5/2_ and Eu 4d_3/2_ orbitals, respectively, confirming the Eu^3+^ oxidation state. A similar trend was observed in Fig. 4F, where the Eu_10%_C7 NPs exhibited distinct sharp, intense peaks at 124.66 eV (*w*_2_) and 121.09 eV (*w*_1_), further confirming the presence of Eu^3+^. Typically, Eu 4d binding energies range from ∼148–125 eV.^38^ Herein, the observed downward shift to ∼128–102 eV was attributed to the substitution of Ce^4+^ by Eu^3+^ within the CeO_2−*x*_ lattice. This caused an electronic structural change due to the formation of Eu–O–Ce bonds. The overlap of Ce 4d in the Eu 4d signals further confirms lattice incorporation, which is driven by hybridization effects. The Ce 4d binding energy range of ∼115–105 eV^39^ reflects the mixed-valence nature of cerium in the Eu_2%_C7 NPs and Eu_10%_C7 NPs. For Eu_2%_C7 NPs, peaks at 111.33 eV (*z*_2_) and 107.85 eV (*z*_1_) are attributed to Ce^3+^–V_o_ interactions and Ce^4+^ states, respectively. Similarly, Eu_10%_C7 NPs exhibited peaks at 113.91 eV (*z*_3_), corresponding to a Ce^3+^ satellite peak, 111.66 eV (*z*_2_) related to Ce^3+^–V_o_ interactions, and 107.51 eV (*z*_1_) associated with Ce^4+^, characterized by a sharper peak. These results confirm the coexistence of Ce^3+^/Ce^4+^ oxidation states, which are maintained after Eu doping, demonstrating the redox flexibility of Eu_*x*%_Cy NPs synthesized *via* combustion doping. The increase in Ce 4d peak intensity with Eu^3+^ doping was due to the enhanced stabilization of Ce^4+^ ions. When Eu^3+^ substitutes Ce^4+^ within the lattice, it suppresses the formation of V_o_—an effect that promotes Ce^3+^ formation. Consequently, the lattice becomes oxidized, resulting in stronger Ce^4+^ signals in the XPS spectra. Analysis of the relative Eu^3+^ content from the integrated peak areas revealed a 59.1% proportion of Eu_2%_C7 NPs, while Eu_10%_C7 NPs exhibited 46.8%. This reduction was due to surface saturation effects, where thermal treatment promotes Eu diffusion deeper into the lattice, resulting in a lower apparent surface concentration.

### 3.4 Effect of antioxidant activity

Fig. S7D† shows the % antioxidant activity of C2, C7, Eu_2%_C7 and Eu_10%_C7 NPs across varying concentrations (5 mg, 2.5 mg, 1.25 mg, 0.625 mg, and 0.3125 mg). At lower concentrations, C2 NPs exhibited radical scavenging capacity, achieving 77.97% activity at 1.25 mg and 71.20% at 0.625 mg. However, at higher concentrations (5 mg and 2.5 mg), a pro-oxidant effect emerged, with negative antioxidant values of −33.27% and −69.56%, respectively. This negative shift in antioxidant activity was observed across all samples at 5 mg, indicating pro-oxidant behavior. This effect results from the oversaturation of free radicals, which exceeds the redox cycling capacity and induces oxidative stress.^12^ At 5 mg, the redox capacity was exceeded, leading to oxidative stress regardless of structural doping. This highlights the concentration-dependent limitation of the redox cycle. A higher surface Ce^3+^ content in C2 NPs (2.08, Ce^3+^/Ce^4+^ ratio) showed radical scavenging at low doses, leading to redox imbalance and destabilization at elevated concentrations. Thermally treated C7 NPs showed scavenging activity at higher concentrations, achieving 72.55% at 2.5 mg, 71.27% at 1.25 mg, and 64.88% at 0.625 mg. This consistency was due to a balanced Ce^3+^/Ce^4+^ ratio (1.62) and moderate *V*_o_, facilitating effective redox cycling with minimized pro-oxidant effects. Eu doping further enhanced the redox stability by promoting Ce^4+^ state stabilization with reduced *V*_o_. The Eu_2%_C7 NPs showed robust antioxidant activity across all concentrations, reaching 86.16% at 2.5 mg, 80.37% at 1.25 mg, and 60.79% at 0.625 mg. In Eu_10%_C7 NPs, antioxidant efficiency remained consistently high, with values of 88.65% (2.5 mg), 89.03% (1.25 mg), and 88.91% (0.625 mg). Even at 0.3125 mg, Eu_10%_C7 NPs maintained activity of 89.97%, reflecting enhanced Ce^4+^ stabilization and superior redox cycling efficiency, even with a lower Ce^3+^/Ce^4+^ surface ratio (0.83). This stabilization prevents ROS accumulation and maintains effective free radical scavenging due to a balanced redox cycle and structural stability. Thus, the combustion synthesis method coupled with post-synthesis thermal refinement effectively promotes *V*_o_ formation and improves the crystalline structure, leading to enhanced antioxidant properties compared to other synthesis methods. This improved redox behavior of Eu_10%_C7 NPs contributes to improved biocompatibility and reduced cellular damage.

### 3.5 Optical properties

#### 3.5.1 Absorbance

The UV-vis spectra exhibited characteristic absorption peaks at 282 and 325 nm, corresponding to the Ce^3+^ ion f-d orbital transitions, indicative of the active redox sites in CeO_2−*x*_ NPs. Notably, the diminished absorbance in the 350–400 nm range suggests a lower concentration of Ce^4+^ ions relative to Ce^3+^ ions, highlighting the presence of *V*_o_ and the mixed-valence nature of CeO_2−*x*_ (Fig. S5A†). A blue shift in the absorption edge was observed with increasing thermal treatment, suggesting improved crystallinity and reduced defect density, consistent with previous literature reports.^40^ Conversely, a red shift in the absorption peak for the Eu_10%_C7 NPs was due to lattice distortion and defect formation induced by Eu^3+^ incorporation, which can alter the electronic environment and promote the stabilization of Ce^4+^ states. Tauc plot analysis, based on direct bandgap transitions (Fig. S5B†), reveals a size-dependent variation in the optical energy bandgap (*E*_g_). Studies have reported that low Eu doping levels (0.25–1 mol%) decrease *E*_g_ to ∼2.75 eV, whereas higher doping levels (2 mol%) can lead to a slight increase (∼3.25 eV).^41^ This variation is attributed to the formation of Eu 4f states below the conduction band edge, modifying the electronic structure and extending absorption into the visible region.^42^ In this study, the combustion-synthesized NPs exhibited reduced *E*_g_ values from 2.61 eV to 2.94 eV. The undoped C2 NPs showed the lowest *E*_g_ of 2.61 eV, which was attributed to a higher Ce^3+^ content and the high presence of *V*_o_, whereas the thermally treated C7 NPs exhibited an increased *E*_g_ of 2.92 eV, which was attributed to enhanced crystallinity and the reduction of defect states. For the Eu-doped samples, Eu_2%_C7 NPs and Eu_10%_C7 NPs exhibited *E*_g_ values of 2.88 eV and 2.94 eV, respectively. Eu^3+^ ions possessed characteristic energy levels within the bandgap of CeO_2−*x*_ NPs, which were associated with their partially filled 4f electron shell. Thus, a slight bandgap widening with Eu doping was observed due to the lattice strain and electronic structure alteration, thereby influencing the charge transfer.

#### 3.5.2 Photoluminescence (PL)

**Fig. 5:**
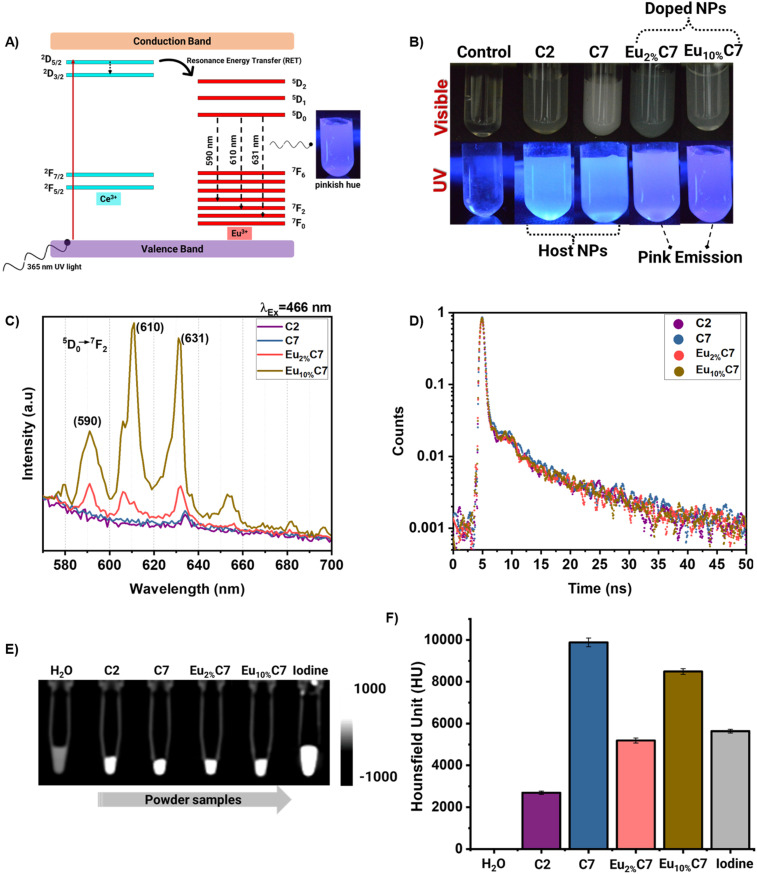
(A) Schematic representation of the energy transfer mechanism in the EC NPs under UV. (B) Photographic images of the doped (Eu_2%_C7 and Eu_10%_C7 NPs) and undoped (C2 and C7 NPs) under UV-A (365 nm, lamp) excitation, showing pink emission. (C) Photoluminescence (PL) spectra of the Eu_2%_C7 and Eu_10%_C7 NPs, exhibiting intense emission peaks at 590, 610, and 631 nm upon excitation at 466 nm. (D) Fluorescence lifetime decay curves of the doped and undoped NPs, with an inset table summarizing fluorescence lifetimes (ns). (E–F) Computed tomography (CT) images and X-ray attenuation intensities (HU) of the doped and undoped NPs, highlighting enhanced contrast properties with NPs compared to the Omnipaque™ iodine contrast agent.

Upon UV-A lamp exposure, Eu^3+^-doped NPs (Eu_2%_C7 and Eu_10%_C7 NPs) displayed a distinct pinkish-red luminescence, attributable to the 4f-4f transitions^43^ of Eu^3+^ ions (Fig. 5B). This visible emission originates from energy transfer processes involving the CeO_2−*x*_ host lattice, where *V*_o_ and Ce^3+^ centers play a critical role in modulating local symmetry and facilitating resonance energy transfer (RET) to Eu^3+^ dopants. No luminescence was observed in the undoped control samples (C2 and C7 NPs), confirming the essential role of Eu^3+^ ions in the observed optical behavior. To understand the excitation-wavelength-dependent spectral responses, the PL spectra were recorded under two excitation conditions. Under 397 nm excitation (Fig. S8C†), broader emission bands were observed—particularly for Eu_10%_C7 NPs—indicating increased spectral overlap due to inhomogeneous broadening. This broadening is attributed to the indirect excitation of Eu^3+^*via* Ce^3+^ 4f-5d transitions and subsequent RET to Eu^3+^ sites in distorted, non-centrosymmetric environments. The excitation at 397 nm primarily engages host-related transitions, thereby exciting a heterogeneous distribution of Eu^3+^ sites, leading to wider hypersensitive transitions and less resolved emission features.

In contrast, under 466 nm excitation (Fig. 5C), Eu_10%_C7 NPs exhibited sharp and well-resolved emission peaks at 590, 610, and 630 nm, corresponding to the ^5^D_0_ → ^7^F_1_, ^5^D_0_ → ^7^F_2_, and ^5^D_0_ → ^7^F_3_ transitions of Eu^3+^, respectively (Fig. 5A). Among these, the ^5^D_0_ → ^7^F_2_ transition—being hypersensitive to site asymmetry—displayed notably higher intensity, indicative of Eu^3+^ ions residing in non-centrosymmetric environments. The 466 nm excitation directly promotes Eu^3+^ from the ^7^F_0_ ground state, resulting in site-specific, symmetry-reflective emissions with enhanced resolution. Thus, the enhanced emission intensity of Eu_10%_C7 NPs compared to Eu_2%_C7 NPs was due to higher Eu^3+^ doping, which increases the density of *V*_o_ (Fig. S8A and B†). Upon excitation, these vacancies disturb the local crystal structure and facilitate electric dipole transitions. Therefore, Eu_10%_C7 NPs with 466 nm excitation and corresponding emission (590–631 nm) are particularly relevant for preclinical optical imaging platforms, such as the IVIS system. Although these hybrid NPs are not suitable for deep-tissue optical imaging, they are ideal for *in vitro* assays and small-animal models, where direct excitation and emission in the visible range are effectively utilized.

#### 3.5.3 PL lifetime analysis

Fig. 5D shows the non-exponential decay with the average lifetimes (*τ*) of C2 NPs (5.827 ns), C7 NPs (5.739 ns), Eu_2%_C7 NPs (5.137 ns), and Eu_10%_C7 NPs (6.037 ns), indicating multi-center emission behavior. These decay profiles were effectively modeled using a multi-exponential function, reflecting the underlying energy transfer dynamics. Generally, two types of Eu^3+^ centers were identified: cubic and perturbed centers. Cubic centers, characterized by high symmetry and eightfold Eu^3+^–O coordination, showed longer lifetimes (0.9–1.2 ms) with minimal quenching due to low radiative transition probabilities.^44^ The perturbed centers influenced by *V*_o_ and local asymmetry displayed shorter lifetimes in ns corresponding to enhanced radiative transition probabilities.^45^ Herein, the increasing Eu^3+^ doping led to a higher density of *V*_o_, intensifying the emissions from the perturbed centers. The longest lifetime was observed for Eu_10%_C7 NPs (6.037 ns), indicating efficient energy transfer and emission site stabilization. The Eu_2%_C7 NPs exhibited a shorter lifetime (5.137 ns), likely due to limited charge transfer efficiency at lower Eu^3+^ concentrations. Additionally, the reduction in *V*_o_ further contributed to the decreased lifetime, making it shorter than that of the host CeO_2−*x*_, C2 NPs (5.827 ns), and C7 NPs (5.739 ns). Emission primarily originates from magnetic dipole (MD) transitions in cubic centers and electric dipole (ED) transitions in perturbed centers.^46^ The intensified ED transitions observed in Eu_10%_C7 NPs synthesized by combustion synthesis emphasize the role of local asymmetry and *V*_o_ in modulating optical properties.

### 3.6 Evaluation of X-ray CT attenuation

CT attenuation measurements were conducted using an X-ray tube Voltage of 80 kV, a tube current of 95 mA, and a slice thickness of 0.6 mm. The results demonstrated significant differences in Hounsfield Unit (HU) (Fig. 5E and F) values across synthesized C2, C7, Eu_2%_C7, and Eu_10%_C7 NPs with controls as water and Omnipaque™ Iodine contrast agents. The reference materials exhibited attenuation values within the ranges: distilled water (0.25 ± 0.02 HU), air (−1005 to −970 HU), polyethylene (−107 to −84 HU), and acrylic (110 to 135 HU), consistent with the standard calibration values for GE BrightSpeed CT systems.^47^ Omnipaque™ Iodine at a concentration of 5 mg demonstrated an attenuation of 5637.52 ± 87.76 HU, aligning with the expected range for iodine-based contrast agents (2000–4000 HU at 100–120 kVp).^48^ The enhanced attenuation at 80 kVp was attributed to the effective photoelectric absorption at its K-edge energy (33.2 keV), resulting in values approaching ∼5600 HU. X-ray scanner-specific factors also contributed to this variation, as reported in GE Optima scanner quality control assessments.^49^ The thermally treated C7 NP host exhibited the highest attenuation of 9881.22 ± 210.40 HU compared with the C2 NPs of 2690.36 ± 79.38 HU. This value is consistent with theoretical predictions based on Ce (*Z* = 58) and its mass attenuation coefficient (MAC) of 2.45 cm^2^ g^−1^ at 100 keV.^50^ The overlap of the X-ray photon energy range at 80 kVp with cerium's K-edge (40.4 keV) and enhanced photoelectric absorption contributes to the high observed HU values. Comparative analysis with high-*Z* contrast agents gold NPs (Au NPs, *Z* = 79), exhibited attenuation values exceeding 5000 HU at similar concentrations due to their strong photoelectric absorption.^51^ In contrast, tantalum-based NPs (*Z* = 73), reported attenuation values ranging from 4000 to 6000 HU.^6^ Herein, Eu_10%_C7 NPs recorded an attenuation of 8490.11 ± 135.12 HU, which was slightly lower than that of undoped C7. The reduction in X-ray attenuation observed with Eu doping was due to changes in charge distribution, which decreased cerium's direct contribution to attenuation. In addition, Eu's K-edge energy (48.5 keV) is less efficient to be excited at 80 kVp with the MAC of 3.04 cm^2^ g^−1^ at 100 keV^52^ compared to cerium. However, the hybridization of these materials enhances attenuation at low X-ray energies, resulting in a higher HU value than Omnipaque™. Eu_2%_C7 NPs exhibited a moderate attenuation of 5188.45 ± 119.68 HU, which is higher than that of C2 NPs, confirming that Eu incorporation enhances attenuation. However, the lower defect density of 2% Eu led to a lower HU value compared to C7 and Eu_10%_C7 NPs. The high attenuation of combustion-synthesized Eu_*x*%_C NPs (doped and undoped) highlights their potential as advanced contrast agents. With HU values between ∼5000 and 9000, they exceed the 2000 HU threshold for dense bone, enabling clear differentiation in X-ray imaging. Therefore, the synthesized NP surface allows seamless integration into the polymer matrix while maintaining higher attenuation than that of commercial contrast agents like Omnipaque™.

Based on physicochemical characterization, C7 and Eu_10%_C7 NPs were incorporated into Hyalgan® to develop CartiOxgel, an IA gel for imaging.

### 3.7 Characterization of the CartiOxgel formulations

**Fig. 6:**
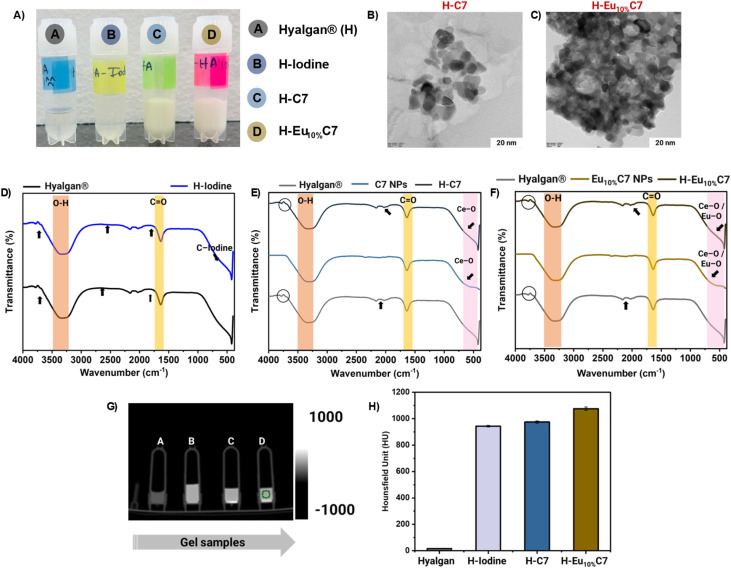
(A) Representative images of CartiOxgel formulations (H–C7 and H-Eu10%C7) alongside controls (Hyalgan® and H-Iodine). (B and C) HR-TEM images illustrating the impregnation of the C7 and Eu_10%_C7 NPs within the Hyalgan® matrix. Scale bar = 20 nm (D–F) Fourier-transform infrared (FTIR) spectra: (D) controls, (E) comparison of C7 NP impregnation in Hyalgan® (H–C7), and (F) comparison of Eu_10%_C7 NP impregnation in Hyalgan® (H–Eu_10%_C7). (G and H) Computed tomography (CT) images and attenuation rates (HU) of the CartiOxgel formulations and controls, demonstrating enhanced contrast properties of the CartiOxgel compared to H-Iodine.

Impregnation of C7 NPs and Eu_10%_C7 NPs into the Hyalgan® gel matrix resulted in the formation of an opaque, gel-like material, particularly noticeable at higher NP concentrations (20 mg mL^−1^), as shown in Fig. 6A. The original transparent nature of Hyalgan® visibly changed to a white, gel-like appearance due to the increased NP content. Highly loaded NPs indicate a potential increase in particle–particle interactions and partial clustering within the matrix. Despite the clear opacity, no sedimentation or phase separation was observed, suggesting that the NPs remained well-incorporated in the gel. The natural hydroxyl and carboxyl groups of HA interact well with the metal-oxide NP surfaces, resulting in stable and uniform dispersions. Compared with dispersions in aqueous ethanol, Hyalgan® provided a stable environment, even without prior surface modification of the NPs. HR-TEM analysis confirmed the successful incorporation of C7 NPs (Fig. 6B) and Eu_10%_C7 NPs (Fig. 6C) within the Hyalgan® gel matrix. NPs are visible, embedded within the polymeric background of the gel. Eu_10%_C7 NPs exhibited localized clustering, likely due to electrostatic interactions between the positively charged particle surfaces and negatively charged HA chains (Fig. S5B†). In contrast, the negative charge of C7 NPs was stabilized by weaker van der Waals interactions (Fig. S5A†). The distinct visibility of individual particles within the matrix supports effective NP dispersion and structural integration into the gel.

Fig. 6D–F with FTIR spectra confirming the chemical interaction between Hyalgan® and NPs (C7 and Eu_10%_C7 NPs). The O–H stretching (3200–3500 cm^−1^) is consistent with the hydroxyl-rich nature of HA.^53^ However, a notable decrease in intensity was observed in H–C7 and H–Eu_10%_C7 compared with their bare counterparts. This difference was due to the hydrogen bonding interactions between the hydroxyl group of Hyalgan® and the surface hydroxyl groups of the NPs. The characteristic peaks at 1500–1700 cm^−1^ were C=O stretching peaks that were present across the Hyalgan®, H–C7, and H–Eu_10%_C7 spectra, reflecting the HA polysaccharide backbone. These peaks were slightly broadened in Eu_10%_C7 NPs compared with those in bare Hyalgan®, indicating the presence of electrostatic interactions between the negatively charged carboxyl groups of HA and the positively charged Eu_10%_C7 NPs. Omnipaque™ iodine usually appears as weak C–I stretches in the fingerprint area (∼500-600 cm^−1^); however, these spectra are not very clear because of the low intensity and spectral overlap. Strong metal–oxygen stretching vibrations (∼500–800 cm^−1^), attributed to Ce–O and Eu–O bonds,^28^ were observed in the C7 and Eu_10%_C7 NPs. In H–C7 and H–Eu_10%_C7, these peaks exhibited reduced intensity, indicating the dispersion of NPs within the gel matrix. These shifts and intensity variations confirm electrostatic interactions and O–H bonding, stabilizing the dispersed NP-based CartiOxgel network.

Fig. 6G shows the CT images of CartiOxgel formulations, while Fig. 6H presents the quantitative analysis of CT attenuation. A significant reduction in X-ray attenuation was observed in the CartiOxgel formulations compared with the bare NPs. The attenuation values for bare C7 NPs (9881.22 ± 210.40 HU) and Eu_10%_C7 NPs (8490.11 ± 135.12 HU) were substantially higher than those of H–C7 (1944.93 ± 207.11 HU) and H–Eu_10%_C7 (1789.38 ± 104.71 HU). This reduction was attributed to the dispersion in a gel matrix, with NPs being spatially separated, reducing the probability of coherent X-ray interaction and thus lowering the overall attenuation.^54^ In the powder form of NPs, their dense packing and high electron density enhance X-ray absorption and scattering, leading to a higher attenuation rate.^55,56^ This aligns with literature reports showing that hydrogel-based nanocomposites reduce X-ray attenuation due to matrix hydration and particle separation.^57^ The attenuation of H–I (898.15 ± 182.14 HU) was significantly lower than that of bare iodine (5637.52 ± 87.76 HU), reinforcing the role of the gel matrix in modulating X-ray contrast. At lower clinical X-ray settings, CartiOxgel remains an effective contrast agent compared with traditional iodine-based formulations, even after incorporation into the Hyalgan®.

### 3.8 *In vitro* cell viability

**Fig. 7:**
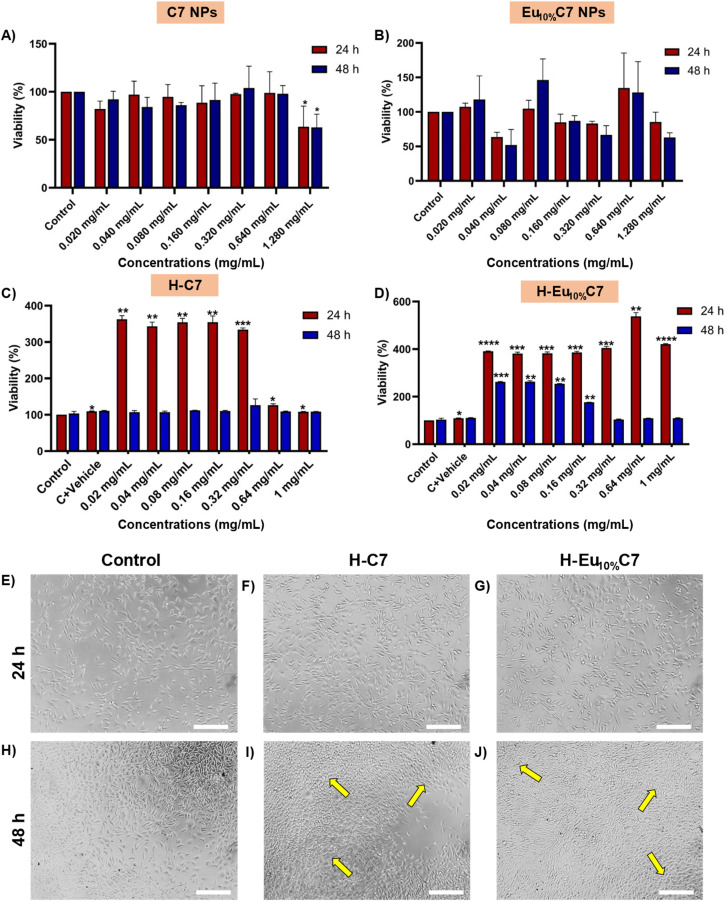
MTT assay of the NIH 3T3 mouse embryonic fibroblast cell lines with the NPs (C7 and Eu_10%_C7 NPs) and the CartiOxgel (H–C7, H–Eu_10%_C7). (A) Cell viability of the C7 NPs at varying concentrations (0.020–1.280 mg) after 24 h and 48 h incubation. A significant reduction in viability was observed at 1.280 mg for both time points (*p < 0.05), while other concentrations showed no significant difference (*p* > 0.05). (B) Cell viability of the Eu_10%_C7 NPs under the same conditions showed no significant cytotoxicity at any concentration (*p* > 0.05). (C and D) Cell viability of the CartiOxgel formulations (H–C7 and H–Eu_10%_C7) at concentrations of 0.020–1.0 mg after 24 h and 48 h. H–C7 showed a significant increase in viability at 24 h across multiple concentrations (*p* < 0.05), with no significant changes at 48 h. H–Eu_10%_C7 exhibited enhanced viability at 24 h across all concentrations (****p < 0.0001 to *p < 0.05) and remained significantly elevated at lower concentrations at 48 h. (E–J) Phase-contrast microscopic images illustrating cell morphology after 24 h (E–G) and 48 h (H–J) exposure of CartiOxgel, showing ∼80% and >100% confluency, respectively, compared to the control. Images were acquired at 10× magnification; scale bar = 100 μm. Data are presented as mean ± SD, and statistical analysis was performed using one-way ANOVA followed by post hoc comparisons.

To determine the optimal concentration of CartiOxgel (H–C7 and H–Eu_10%_C7) for live animal imaging applications, the biocompatibility of the formulations was evaluated using fibroblast cells (Fig. 7). Cell viability was assessed at 24- and 48-h following treatment with varying concentrations of C7, Eu_10%_C7 NPs, Hyalgan® (vehicle), H–C7, and H–Eu_10%_C7, using the MTT assay (Fig. S8†). Previous studies reported the dose-dependent cytotoxicity of EC and CeO_2−*x*_ NPs, highlighting their potential role against oxidative stress-induced cellular damage.^15,58^ Consistent with these findings, our combustion-synthesized C7 NPs at 24 and 48 h showed a significant reduction (*p* = 0.0174, 0.0149) in cell viability at a higher dose (1.28 mg mL^−1^) with 63.5% ± 21.49% and 62.9% ± 13.7%, respectively (Fig. 6A). However, for the same periods, H–C7 mitigated the cytotoxicity with significantly higher cell viability at 1 mg mL^−1^ (108.9% ± 0.002% and 108.6% ± 0.001%) and across all tested concentrations (*p* > 0.05) (Fig. 6C). Control + vehicle (Hyalgan®) provides a protective microenvironment that reduces direct NP-cell interactions and mitigates toxicity.^59,60^ Its proliferative and anti-inflammatory properties enhance cell adhesion, migration, and extracellular matrix remodeling. Similarly, Eu_10%_C7 NPs increased cell viability to 85.3% ± 14.17% after 24 h but declined to 63.02% ± 6.7% at 48 h. This reduction after prolonged NP exposure was due to elevated intracellular ROS, which disrupted cellular homeostasis and induced apoptosis. However, H–Eu_10%_C7 maintained exceptionally high viability at both time points (24 h: 423.78% ± 0.02%; 48 h: 110.01% ± 0.01%) with no significant reductions observed at any dose (*p* > 0.05). The combination of bare NPs with Hyalgan® mitigated oxidative stress-induced cytotoxicity, promoting cell survival and thus enhancing fibroblast confluence, which was observed even after 48 h (Fig. 6E–J). The hydrogel's sustained release mechanism led to a significant increase in viability (>300%) at lower concentrations within 24 h. Over time, the gradual release of NPs resulted in a decline in cell viability; however, in the presence of Hyalgan®, cell viability remained at 100% even at higher concentrations, demonstrating the protective effect of Hyalgan®. The half-maximal inhibitory concentration (IC_50_) values, derived from the dose–response curves, confirm enhanced biocompatibility. At 24 h, C7 exhibited an IC_50_ of 0.98 mg mL^−1^, whereas H–C7 increased to 1.45 mg mL^−1^. By 48 h, IC_50_ values were 0.88 mg mL^−1^ for C7 and 1.55 mg mL^−1^ for H–C7. Similarly, Eu_10%_C7 NPs showed an IC_50_ of 1.12 mg mL^−1^ at 24 h, reflecting their high antioxidant activity, whereas H–Eu_10%_C7 increased to 1.50 mg. At 48 h, Eu_10%_C7 NPs and H–Eu_10%_C7 exhibited IC_50_ values of 1.05 and 1.60 mg mL^−1^, respectively. After 24 h of exposure, the ROS scavenging ability of C7 NPs induced a pro-oxidant effect due to fluctuations caused by the elevated Ce^3+^/Ce^4+^ ratio. The lower Ce^3+^/Ce^4+^ ratio in Eu_10%_C7 NPs minimized the pro-oxidant transition, resulting in a more stable IC_50_ value over time. For live animal imaging, IC_70_ values were assessed to achieve optimal imaging with minimal toxicity. H–C7 exhibited IC_70_ values of 1.80 mg mL^−1^ at 24 h and 1.90 mg mL^−1^ at 48 h, while H–Eu_10%_C7 recorded IC_70_ values of 1.85 mg mL^−1^ and 1.95 mg mL^−1^, respectively.

### 3.9 *In vivo* CT imaging of the rat knee region

**Fig. 8:**
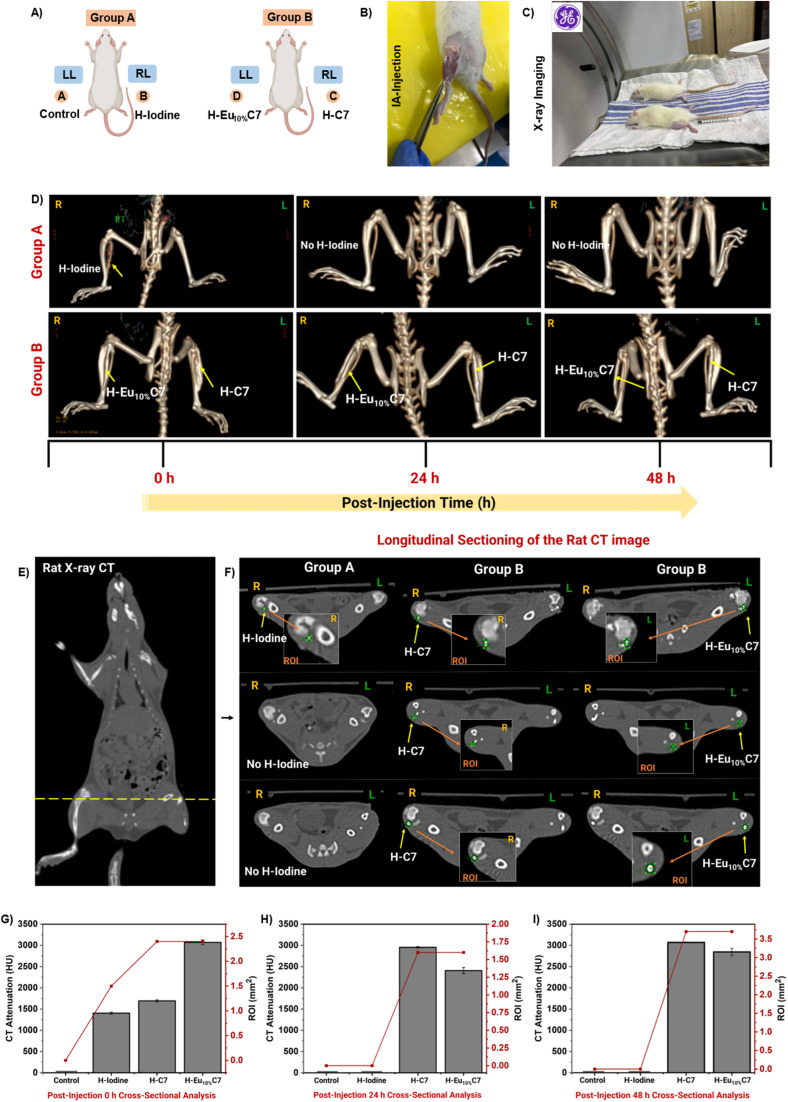
*In vivo* CT imaging of the knee region in IA-injected rats administered with H-Iodine and the CartiOxgel (H–C7, H–Eu_10%_C7). (A) Schematic representation of the experimental groups: Group A—left leg (LL) as the control and right leg (RL) injected with H-Iodine; Group B—LL injected with H–Eu_10%_C7 and RL with H–C7. (B) Anesthetized rat with a shaved knee region, confirming no external leakage post-IA injection. (C) Positioning of the rat in the scan bed for X-ray imaging using a GE Optima CT 64-slice scanner. (D) 3D volume-rendered CT reconstruction of the knee joints in Groups A and B at 0, 24, and 48 h post-injection, with arrows indicating IA-injected H-Iodine and CartiOxgel. (E) Whole-body 3D CT scan of the rat, with a yellow line marking the cross-section of the knee region. (F) 3D cross-sectional CT visualization of the knee, highlighting the region of interest (ROI, green circle) used for CT attenuation measurements (HU) over time. (G–I) Quantitative CT attenuation values from the selected ROI, illustrating contrast retention and dissipation dynamics over 0 h, 24 h, and 48 h. Data are presented as mean ± SD.

Given the promising attenuation rates and *in vitro* performance of CartiOxgel, we investigated its potential as a CT contrast agent for IA imaging. *In vivo,* CT imaging was conducted in healthy Wistar rats to evaluate the retention and image enhancement of CartiOxgel formulations (H–C7 and H–Eu_10%_C7) in comparison with a commercial Omnipaque™-Iodine-based contrast agent (as H-Iodine). As depicted in Fig. 8A, in Group A, the left leg (LL) served as the control (Hyalgan®), while the right leg (RL) was injected with H-Iodine. In Group B, the LL received H–Eu_10%_C7, and the RL was injected with H–C7 (*n* = 3 per group). Before IA administration, each rat was anesthetized and the knee joint region was shaved to ensure optimal injection conditions. A standardized volume of 50 μL CartiOxgel was carefully delivered into the joint space with careful monitoring (Fig. 8B). Imaging was performed using a CT scanner as shown in Fig. 8C, post-injection (0 h) to verify the localization of the contrast agent within the knee joint. CT imaging demonstrated distinct attenuation profiles among the contrast agents. Advanced 3D reconstruction and VR visualization confirmed that CartiOxgel achieved radiopacity comparable to that of bone (Fig. 8D). At a low concentration (1 mg/0.05 mL), CartiOxgel exhibited superior contrast enhancement over Omnipaque™-Iodine, highlighting its efficacy for IA imaging. Despite a controlled injection method, minor leakage from the synovial joint was observed in the knee region, particularly along the tibia and fibula. Literature reports indicate that IA injection of Iobitridol (30–50 μL) in rats often leads to leakage due to anatomical vulnerabilities.^61,62^ Unlike the human knee with an enclosed joint capsule and an upright orientation, the rat knee is positioned at an acute angle with increased joint mobility, making it more susceptible to fluid dispersion. A key site contributing to leakage is the tendon musculus extensor digitorum longus (EDL), which originates from the lateral condyle of the tibia and crosses the joint capsule and exits through the profundus of the extensor retinaculum.^61^ This anatomical pathway provides a potential escape route for injected fluids, particularly under mechanical stress and limb movement. Variations in joint positioning before and after IA administration further influence fluid distribution, contributing to localized dispersion outside the joint space.

Fig. 8E shows the longitudinal cross-section of the rat X-ray CT, analyzed using GE software, which confirms the spatial distribution of CartiOxgel and provides quantitative CT attenuation measurements (Fig. 8F) based on the Region of Interest (ROI). H-Iodine exhibited an attenuation of 1403 ± 47.60 HU for a limited ROI of 1.5 mm^2^ at 0 h, whereas H–C7 and H–Eu_10%_C7 demonstrated significantly higher attenuation values of 1693 ± 43.13 HU and 3071 ± 101.2 HU, respectively, with an expanded ROI of 2.4 mm^2^. At 24 h post-injection, no detectable contrast region was observed for H-Iodine, indicating its rapid clearance from the joint space, consistent with literature reports on the fast elimination kinetics of iodine-based agents.^3^ CartiOxgel exhibited prolonged retention, with attenuation values of 2955 ± 28.5 HU (H–C7) and 2407 ± 126.23 HU (H–Eu_10%_C7), localized within a 1.6 mm^2^ region. The prolonged retention of CartiOxgel is attributed to strong electrostatic interactions with cartilage GAGs, consistent with findings from nanoparticle-based IA imaging studies.^63^ At 48 h post-injection, the attenuation values increased to 3071 ± 18.73 HU (H–C7) and 2845 ± 148.88 HU (H–Eu_10%_C7), with an expanded localized region of 3.7 mm^2^. This variation in ROI selection was due to the differences in animal positioning and intergroup variability, leading to localized fluctuations in HU values (Fig. 8G–H). The extended retention of CartiOxgel beyond 48 h highlights its potential for prolonged IA imaging applications. The delayed clearance was due to its larger hydrodynamic size, which restricts diffusion from the synovial cavity. Thus, CartiOxgel exhibited superior contrast, prolonged retention, and slower clearance, making it a viable alternative to Omnipaque™ for IA imaging.

### 3.10 *Ex vivo* optical imaging of the rat knee region

**Fig. 9:**
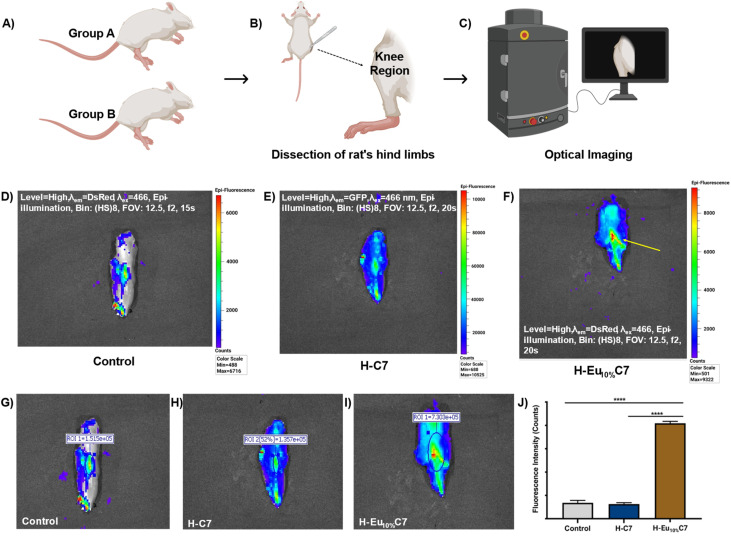
*Ex vivo* optical imaging of the rat knee region at 48 h post-IA injection. (A–C) Schematic representation of the procedure followed after euthanasia for optical imaging. (D–F) Epi-fluorescence imaging of the knee region using IVIS with 466 nm excitation and DsRed emission filter. (G–I) ROI selection for fluorescence intensity quantification using Living Image® software. (J) Total fluorescence intensity of the control, H–C7, and H–Eu_10%_C7 groups, showing a significant increase in the H–Eu_10%_C7 group compared with the control and H–C7 groups (****p < 0.0001). Data are presented as mean ± SD (*n* = 3).

At 48 h post-IA injection, the euthanized rats' limbs (from Group A and Group B) were dissected to examine the emission effects of CartiOxgel on the knee region. Fig. 9 presents the optical imaging of the *ex vivo* rat knee region using the IVIS. The pseudo-color intensity variation (Fig. 9D–F) provides a direct visualization of the presence and distribution of different formulations. The ROI-based intensity measurements revealed that Group A (control) exhibited a total fluorescence count of 1.52 × 10^5^ within an ROI of 0.58 cm^2^ (Fig. 9G). Group B H–C7 showed an intensity of 1.36 × 10^5^ within a reduced ROI of 0.28 cm^2^ (Fig. 9H). Excitation at 466 nm did not produce an emission signal for H–C7 (Fig. 9E). However, as indicated by the red region in Fig. 9F, H–Eu_10%_C7 generated a pseudo-color signal under the same excitation, confirming their detectability in the optical imaging system. This observation underscores that the optical signal detected from the knee region originates from the distinct fluorescence profile of the NPs rather than tissue autofluorescence. H–Eu_10%_C7 exhibited a significant increase in fluorescence, with a total count of 8.06 × 10^5^ within an ROI of 1.97 cm^2^, representing a ∼5.9-fold enhancement compared with the control and H–C7. This substantial increase highlights the superior fluorescence efficiency of Eu_10%_C7 NPs, attributed to their hypersensitive emission in the reddish-pink region (590–631 nm). This enhanced emission facilitates high-sensitivity signal detection while minimizing autofluorescence-related artifacts, thereby improving imaging quality. Conventional IA contrast agents suffer from various issues, for instance, indocyanine green (ICG, *λ*_ex_ = 780 nm, *λ*_em_ = 820 nm),^64^ which suffers from low photostability, and fluorescein sodium (*λ*_ex_ = 490 nm, *λ*_em_ = 510 nm), which exhibits limited tissue penetration and significant scattering within biological matrices. The integration of metal oxides into IA imaging remained limited because of suboptimal fluorescence emission and insufficient tissue penetration. Addressing these challenges, the development of Eu_10%_C7 NP-based CartiOxgel represents a significant advancement in IA optical imaging, offering high-contrast, real-time visualization for knee imaging applications. In addition, its internalization into the articular cartilage was examined to evaluate diffusion efficiency through histological analysis.

### 3.11 Histological evaluation

**Fig. 10:**
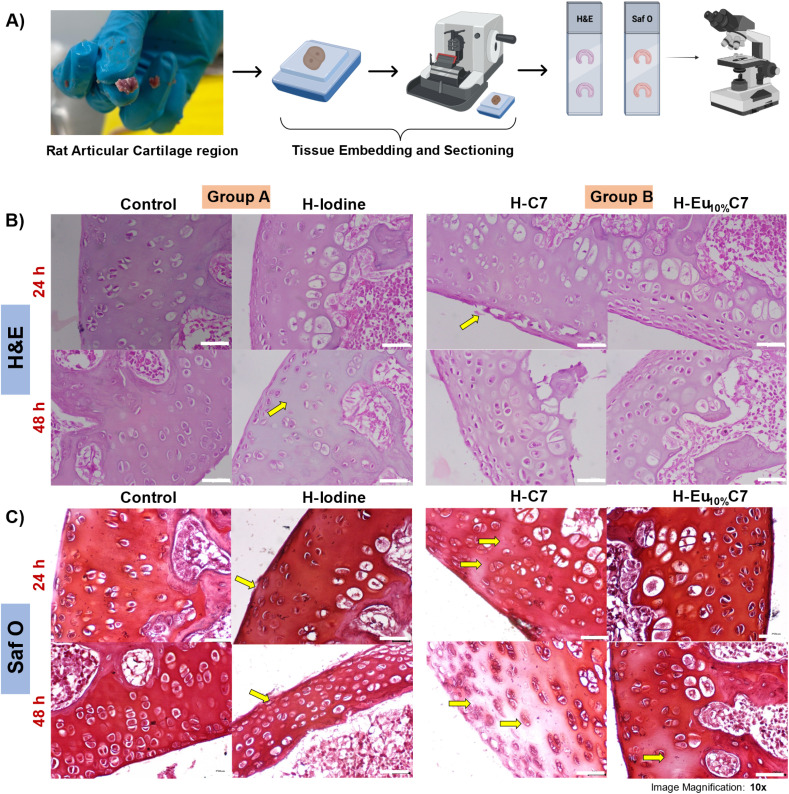
(A) Schematic representation of the procedure for harvesting the articular region of the rat knee, followed by embedding and sectioning for histological analysis. (B and C) Hematoxylin and eosin (H&E) and safranin O (Saf O) staining of Group A (control and H-Iodine) and Group B (H–C7 and H–Eu_10%_C7) at 0 and 24 h post-injection. Images were acquired at 10× magnification; scale bar = 250 μm. The yellow arrows indicate regions with reduced staining intensity, structural alterations, and matrix degradation.

Fig. 10 presents the histological analysis of cartilage tissues stained with hematoxylin and eosin (H&E) and safranin O (Saf O) from Groups A and B at 24 and 48 h post-treatment. These tissue sections, magnified to highlight both cellular and extracellular matrix (ECM) structures, provide insights into morphological changes over time. Given the sub-20 nm size of NPs, this study aimed to assess their cartilage penetration and long-term imaging potential. To quantitatively evaluate the effects of CartiOxgel on cartilage tissue, a modified Mankin scoring method was employed for both H&E and Saf O staining.^65^

#### 3.11.1 Cartilage structure and cellular response

Tissue structure, cellularity, matrix staining, and tidemark integrity were assessed, with a total score ranging from 0 (normal) to 14 (severe degeneration). The H&E staining scores for the CartiOxgel are summarized in Table S3.† At 24 h, H-Iodine and H–Eu_10%_C7 cartilage exhibited uniformly distributed chondrocytes with distinct space and an intact ECM to a Mankin score of 0, indicating no structural damage. However, a noticeable slight disruption in the superficial zone of H–C7 was observed in Fig. 10B (score 1). After 48 h, histological differences became evident for H-Iodine (Fig. 10B) with a noticeable yellow hue in the transition zone due to iodine deposition and altered proteoglycan composition. The high affinity of iodine for organic materials facilitated its diffusion through Hyalgan® into the cartilage, leading to accumulation within the ECM and subsequent alteration of staining properties.^66^ Overall, the mild tidemark integrity loss by chondrocyte condensation indicates that the early-stage ECM remodeling led to the total Mankin score of 5 for H-iodine. H–C7 showed slight alterations in the matrix integrity with mild cellular reorganization and minimal tidemark alterations with a total score of 3, whereas H–Eu_10%_C7 cartilage showed no significant fibrillation or chondrocyte clustering (score 0). The antioxidant properties of Eu_*x*%_C NPs contributed to their cytoprotective effects, mitigating oxidative stress-induced cellular damage. Thus, the H&E Mankin score remained at 0 for H–Eu_10%_C7 post 48 h with negligible cartilage damage compared to the H-Iodine.

#### 3.11.2 GAGs retention and matrix stability

Saf O staining was used to evaluate GAGs content within the cartilage tissue. Table S4† summarizes the staining intensity, homogeneity, and Mankin's scores (0–14). At 24 h, the control exhibited uniform Saf O staining intensity, indicating a structurally intact cartilage matrix (score 0). However, a localized reduction in staining intensity was observed in the H–C7 cartilage, suggesting early interactions between NPs and the ECM (score 2). After 48 h, H-Iodine exhibited a marked reduction in staining intensity, indicative of early GAG depletion, with a Mankin score of 3. H–C7 showed a substantial staining intensity reduction in the transitional zone, implying early GAG loss, with a Mankin score of 6. H–Eu_10%_C7 exhibited minor heterogeneity in staining patterns, with a Mankin score of 1 indicating greater GAG retention and better biocompatibility than H–C7. Thus, the histological findings confirmed the CartiOxgel penetration into the transitional zone, an effective factor for cartilage imaging. The biocompatibility and GAG retention observed in H–Eu_10%_C7 formulated CartiOxgel resulted in better penetration and long-term stability compared to conventional Omnipaque™ iodine-based contrast agents. Furthermore, CartiOxgel demonstrated notable cartilage preservation, with no evidence of chondrocyte clustering, fibrillation, or ECM disintegration, reinforcing its potential as a stable IA contrast agent.

## 4. Conclusion

This study successfully demonstrated the doping of Eu into CeO_2−*x*_ NPs using a one-pot combustion synthesis method, followed by thermal treatment to enhance physicochemical properties. High-temperature annealing at 700 °C not only improved crystallinity and structural stability but also facilitated efficient Eu incorporation, resulting in well-defined nanostructures with distinct material characteristics. The combustion-synthesized NPs (C7, Eu_2%_C7, and Eu_10%_C7 NPs) exhibited an average particle size of ∼20 nm, making them promising candidates for cartilage internalization. The incorporation of Eu^3+^ ions into the Ce lattice resulted in enhanced optical luminescence under UV-A excitation and improved X-ray attenuation, demonstrating their dual imaging potential. The contrast performance of EC NPs surpassed that of commercially available iodine-based contrast agents (Omnipaque™). Furthermore, cell viability assays confirmed excellent biocompatibility even after 48 h of exposure, attributed to the synergistic antioxidant effect of Ce^3+^/Ce^4+^ redox behavior.

To further optimize IA imaging applications, C7 and Eu_10%_C7 NPs were incorporated into Hyalgan®, an FDA-approved IA injectable gel, forming a hybrid contrast agent termed “CartiOxgel”. *In vivo* imaging using clinical X-ray CT demonstrated that CartiOxgel exhibited prolonged retention and a controlled-release profile within the knee joint, maintaining stability up to 48 h post-IA injection. CartiOxgel demonstrated X-ray attenuation comparable to bone, appearing radio-dense even at a low concentration (1 mg/0.05 mL) compared to Omnipaque™-iodine-based contrast agent. The optical luminescence properties of Eu_10%_C7 NPs enabled optical imaging of the knee region using IVIS. The reddish-pink hypersensitive emission (590–631 nm) penetrates through the tissue barriers and provides a prominent luminous signal even 48 h post-IA injection in euthanized rats. Histological analysis confirmed the effective penetration of NPs within the articular cartilage, reaching the transitional zone, indicating effective tissue integration and penetration. These findings establish CartiOxgel as a promising multifunctional IA contrast agent for X-ray CT and optical imaging. Investigations into the diagnostic potential of CartiOxgel in assessing cartilage damage using micro-CT imaging across various stages of cartilage degeneration (from early-stage lesions to severe damage) are currently underway. However, further studies are needed to clarify its systemic biodistribution and clearance to ensure its long-term safety.

## Author contributions

Conceptualization: Ashwin Kumar Narasimhan; methodology: Hema Brindha Masanam and Ashwin Kumar Narasimhan; experimentation: Hema Brindha Masanam and Sina Jafari; formal analysis and investigation: Ashwin Kumar Narasimhan and Hema Brindha Masanam; experimental support: Margaret Salomi, Victor R. Lazar, Senthil Kumar Aiyappan and Priyatha Premnath; writing – original draft preparation: Hema Brindha Masanam; writing – review and editing: Hema Brindha Masanam and Sruthi Ann Alex; supervision: Ashwin Kumar Narasimhan and Sruthi Ann Alex.

## Conflicts of interest

The authors declare that they have no conflicts of interest.

## Supplementary Material

RA-015-D5RA01830G-s001

## Data Availability

The authors confirm that the data supporting the findings of this study are available within the article [and/or] its ESI.†
