# Supplementary material for: Europium-doped cerium oxide nanoparticle-impregnated Hyalgan® (CartiOxgel): an intra-articular contrast agent for X-ray CT and optical imaging
Source: RSC Adv. 2025 Jul 7;15(29):23374–95. doi: 10.1039/d5ra01830g (PMC12230787; doi:10.1039/d5ra01830g)
Supplement: RA-015-D5RA01830G-s001 [file RA-015-D5RA01830G-s001.pdf]

Supplementary Information (SI) for RSC Advances.
This journal is © The Royal Society of Chemistry 2025

Supplementary Information

Europium-Doped Cerium Oxide Nanoparticle-Impregnated Hyalgan® (CartiOxgel): An Intra-Articular Contrast Agent for X-ray CT and Optical Imaging

Hema Brindha Masanam[1], Sina Jafari[2], Ashwin Kumar Narasimhan[2], Priyatha Premanth[2], Margaret Salomi[3], Victor R Lazar[4], Sentil Kumar Aiyappan[4], Sruthi Ann Alex[1]*

[1]*Department of Biomedical Engineering, SRM Institute of Science and Technology, Kattankulathur-603203, Tamil Nadu, India.*
[2]*Department of Biomedical Engineering, College of Engineering and Applied Science, University of Wisconsin, 3200 North Cramer Street, Milwaukee, WI 53211, United States of America.*
[3]*Specific Pathogen Free Animal House (SPF-AH), SRM Institute of Science and Technology, Kattankulathur-603203, Tamil Nadu, India.*
[4]*Department of Radio-Diagnosis, SRM Medical College Hospital and Research Centre, Kattankulathur-603203, Tamil Nadu, India.*
**Corresponding Author Email ID: sruthia@srmist.edu.in*

Table of Content

Item	Title	Page No.
Figure S1	Microstructural Analysis through XRD.	2
Figure S2	HR-TEM images with Fast Fourier Transform (FFT) analysis	3
Figure S3	Scanning transmission electron microscopy (STEM) image	4
Figure S4	Energy-dispersive X-ray (EDX) spectra	4
Table S1	Morphological studies: d (nm), PDI, Zeta potential	4
Figure S5	HR-TEM images of CartiOxgel	5
Figure S6	XPS survey scan and O 1s scan	6
Table S2	Fitted XPS data	7
Figure S7	Antioxidant activity assessment	8
Figure S8	UV-Vis absorption spectra	9
Figure S9	$Eu_{10\%}C7$ NPs luminous Imaging under UV-A excitation	9
Figure S10	Fibroblast cells treated with control + vehicle (Hyalgan®)	10
Figure S11	MTT-stained cells	10
Table S3	Scoring metrics for H&E-stained	11
Table S4	Scoring metrics for Saf O	11

XRD Data:

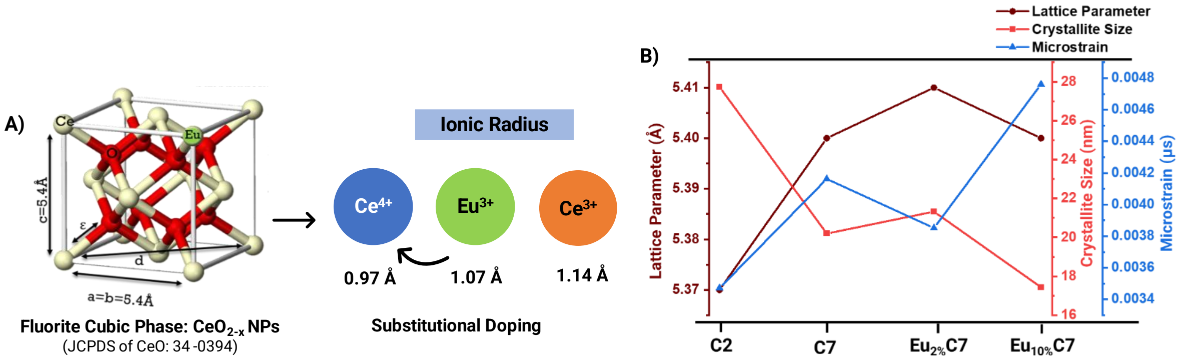

Figure S1. Microstructural Analysis through XRD. A) Schematic representation of the fluorite cubic crystal structure of CeO_{2-x}, with Europium (Eu) substitutionally doped. B) Multiplot displaying the variations in lattice parameter, crystallite size, and microstrain.

TEM Data:

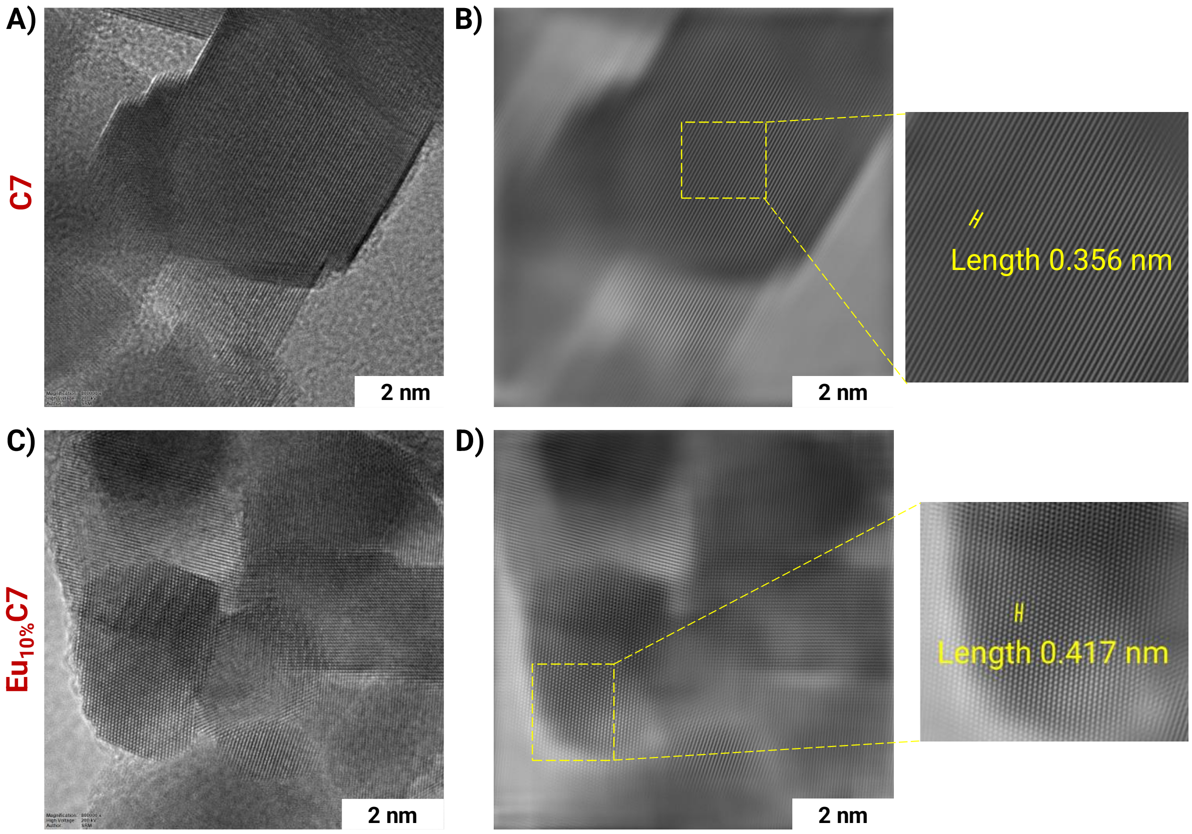

Figure S2. HR-TEM images with Fast Fourier Transform (FFT) analysis for interfacial lattice evaluation. (A–B) C7 nanoparticles showing clear lattice fringes and an interplanar spacing of 0.356 nm. (C–D) $Eu_{10\%}$C7 NPs exhibiting slightly expanded lattice fringes with a spacing of 0.417 nm, suggesting interface strain due to europium doping. All scale bars represent 2 nm.

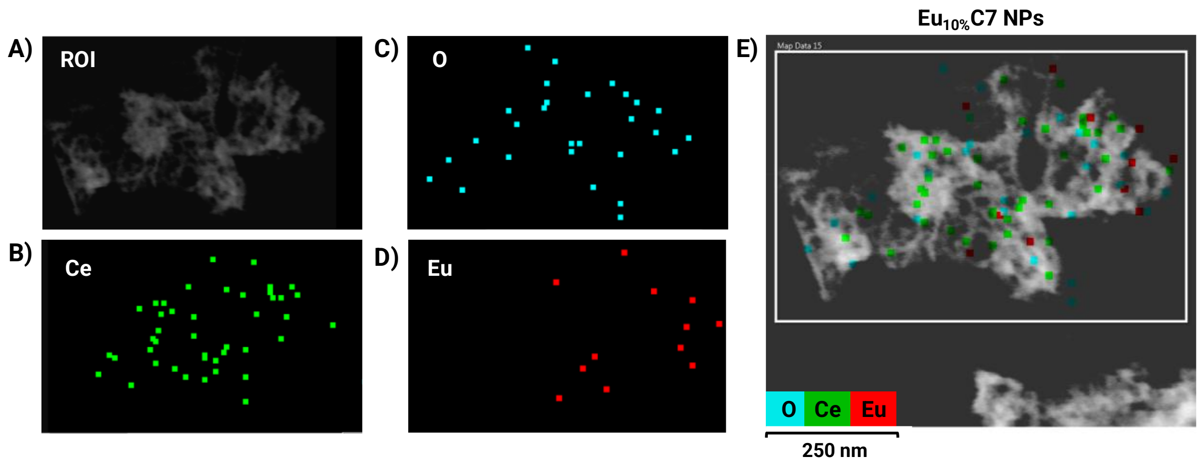

Figure S3. Scanning transmission electron microscopy (STEM) image and elemental mapping of $Eu_{10\%}C7$: (A) Region of interest (ROI) for elemental analysis; (B) cerium (Ce) mapping; (C) oxygen (O) mapping; (D) europium (Eu) mapping; (E) combined elemental map of the ROI.

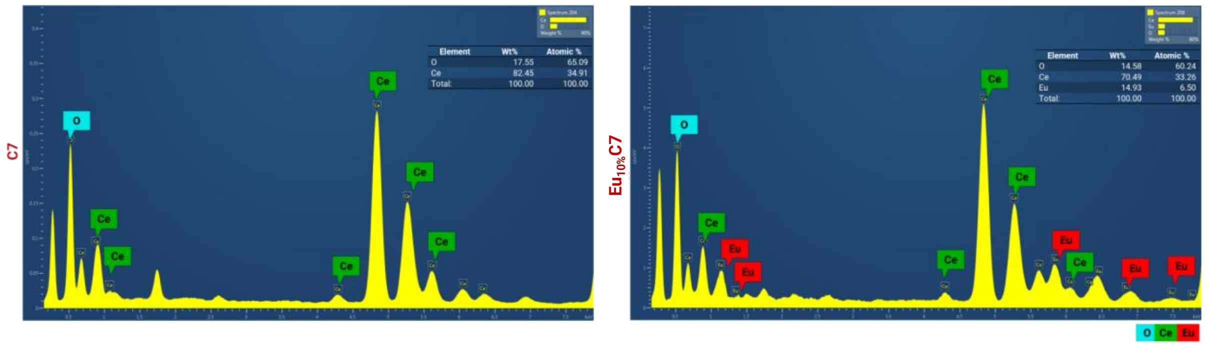

Figure S4. Energy-dispersive X-ray (EDX) spectra of C7 and $Eu_{10\%}C7$ NPs with an inset table showing the elemental weight% and atomic%.

Table S1. Hydrodynamic diameter, polydispersity index (PDI), and zeta potential of the combustion-synthesized nanoparticles (NPs).

Sample	C2	C7	$Eu_{2\%}C7$ NPs	$Eu_{10\%}C7$ NPs
d (nm)	1264 ± 1126	925.9 ± 168.3	1044 ± 136.1	1186 ± 361.3
PDI	0.58	0.519	0.124	0.274
Zeta Potential (mV)	-6.02	-6.51	-11.8	+20.7

*Dispersed Medium: Ethanol

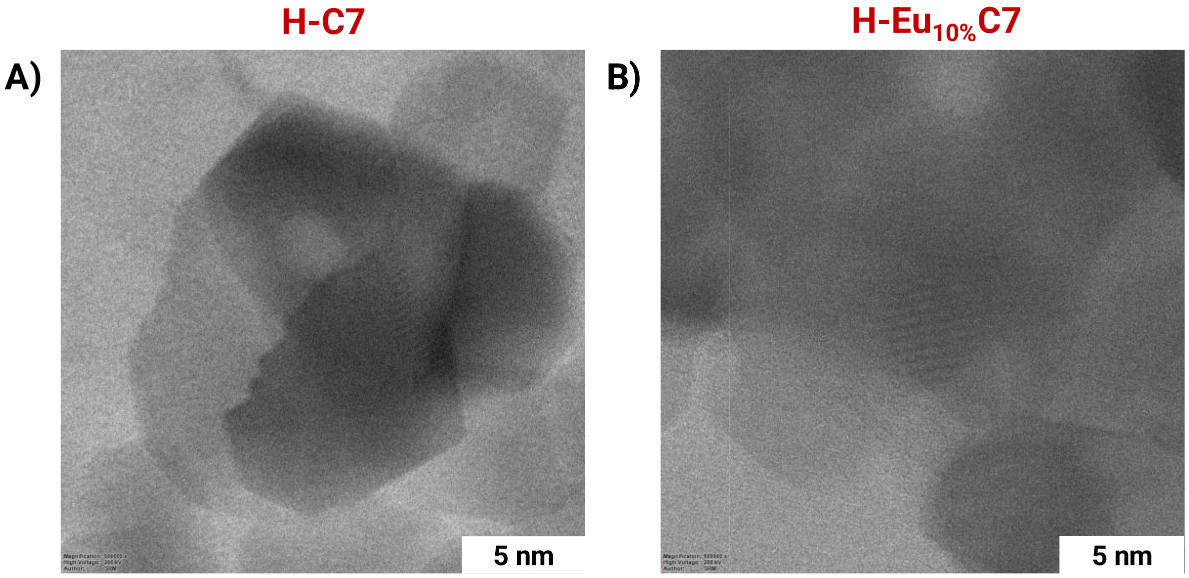

Figure S5. High-resolution transmission electron microscopy (HR-TEM) images of (A) H-C7 with an inset showing the selected area electron diffraction (SAED) pattern and (B) H-$Eu_{10\%}$C7 with an inset showing the SAED pattern. Scale bars= 5 nm (HR-TEM) and 2 1/nm (SAED).

XPS data:

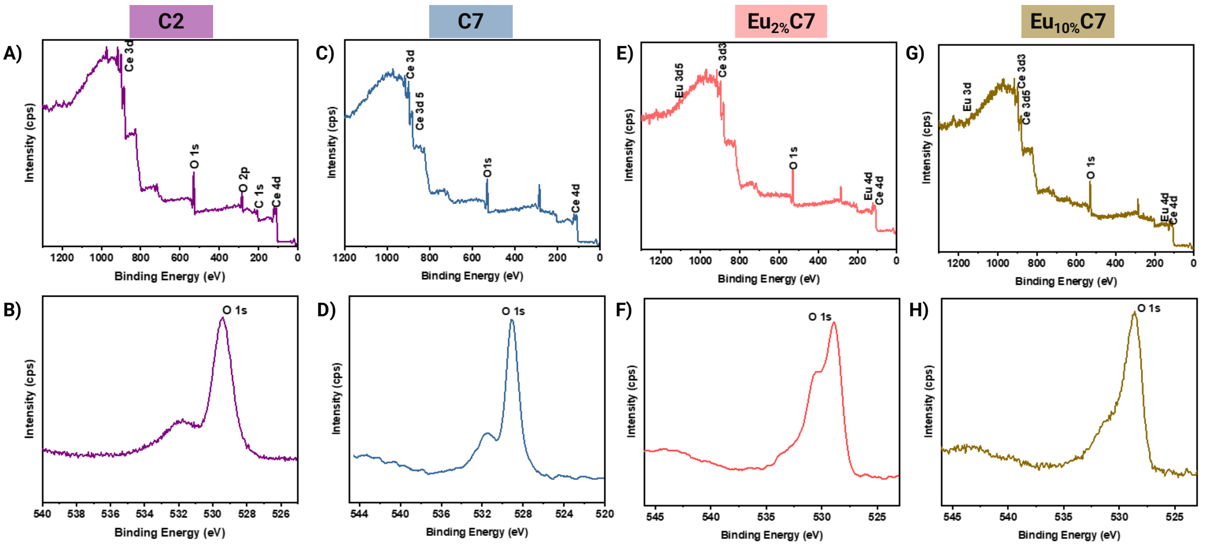

Figure S6. (A–B) XPS survey scan and O 1s scan of C2 NPs; (C–D) XPS survey scan and O 1s scan of C7 NPs; (E–F) XPS survey scan and O 1s scan of $Eu_{2\%}C7$ NPs; (G–H) XPS survey scan and O 1s scan of $Eu_{10\%}C7$ NPs, highlighting the presence of Ce, Eu, and O.

Table S2. The table presents the fitted XPS spectra binding energy values and their corresponding integrated areas of the combustion synthesized NPs.

**C2 NPs**	u_3	u_2	u_1	v_3	v_2	v_1
**Binding Energy (eV)**	916.19	905.429	900.493	897.782	886.674	881.892
**Integrated Area**	7715.01	5224.955	6155.244	10055.119	19921.629	9780.22
**C7 NPs**	u_3	u_2	u_1	v_3	v_2	v_1
**Binding Energy (eV)**	916.795	905.349	901.0126	898.32	886.56	882.417
**Integrated Area**	7089.96	10382.877	7402.32	9783.67	20223.253	10245.5779
**$Eu_{2\%}$C7 NPs**	u_3	u_2	u_1	v_3	v_2	v_1
**Binding Energy (eV)**	916.77	907.9151	899.952	8971.42	887.42	882.4578
**Integrated Area**	11641.88	2097.37	17350.22	4897.83	24003.106	20874.47
	w_2	w_1	z_2	z_1		
**Binding Energy (eV)**	124.54	121.37	111.33	107.854		
**Integrated Area**	4243.35	3125.8503	1239.83	3.867.83		
**$Eu_{10\%}$C7 NPs**	u_3	u_2	u_1	v_3	v_2	v_1
**Binding Energy (eV)**	916.25	907.619	899.938	897.472	886.565	882
**Integrated Area**	11096.596	1876.1432	12120.722	9220.11	13714.9	15000.152
	w_2	w_1	z_3	z_2	z_1	
**Binding Energy (eV)**	124.663	121.091	113.907	111.65861	107.505	
**Integrated Area**	1130.97	2258.53	373.0019	567.892	2913.301	

Antioxidant Assay:

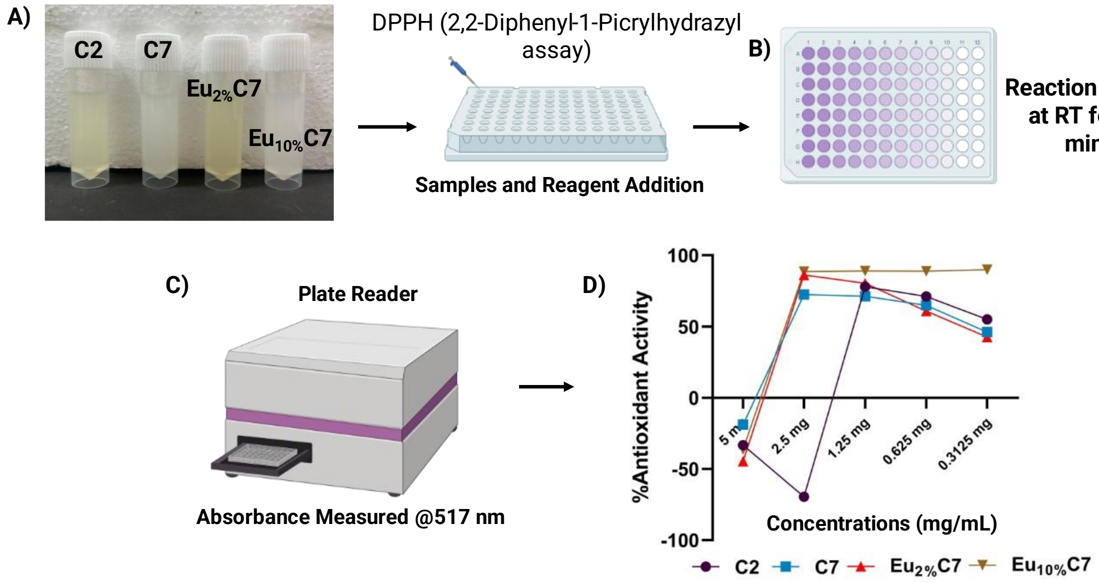

Figure S7. Antioxidant activity assessment of undoped (C2, C7) and doped ($Eu_{2\%}$C7, $Eu_{10\%}$C7) using the DPPH assay. A) NPs samples prepared at concentrations of 5.0, 2.5, 1.25, 0.625, and 0.3125 mg/mL for the assay. B) Reaction setup of NPs with DPPH solution incubated at room temperature for 30 minutes. C) Absorbance measured at 517 nm using a microplate reader. D) Dose-dependent antioxidant activity (%) of the NPs across different concentrations.

Optical Properties:

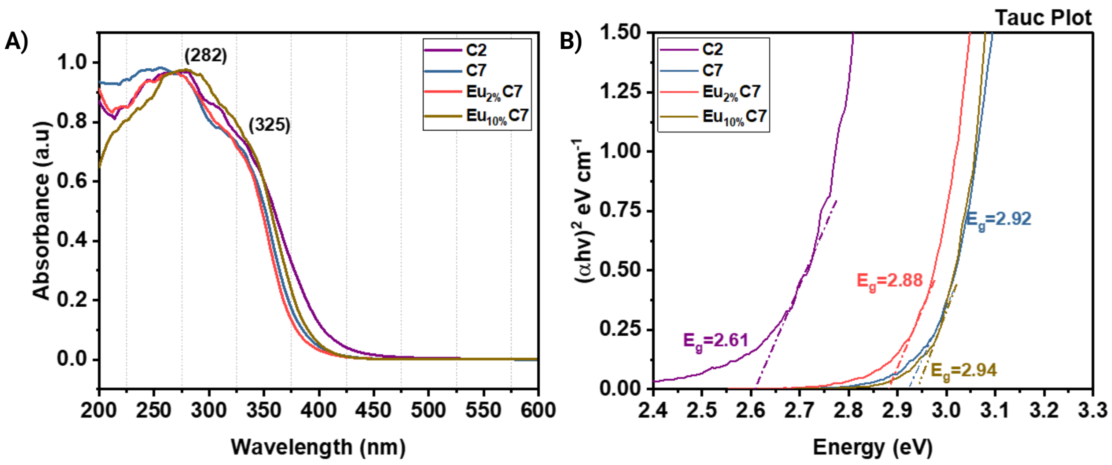

Figure S8. UV-Vis absorption spectra of undoped and doped NPs: (A) Absorbance peaks of the samples; (B) Tauc plot showing linear intercepts on the x-axis, indicating bandgap values ranging from 2.61 eV to 2.94 eV.

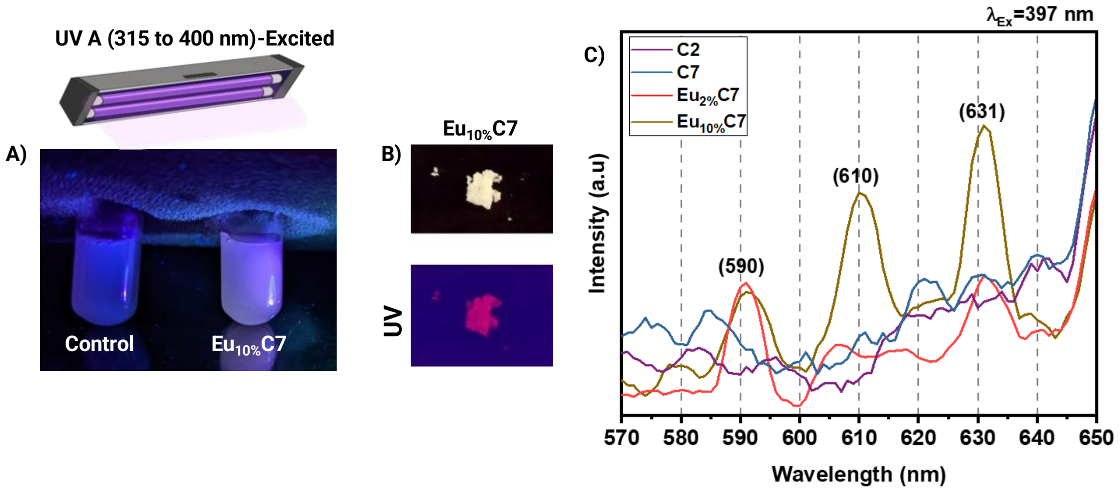

Figure S9. Representative images of combustion-synthesized hybrid NPs ($Eu_{10\%}C7$ NPs) under UV-A exposure. A–B) Enhanced pinkish-red luminescence observed in both liquid suspension and powder form confirms the characteristic photoluminescent behavior of Eu^{3+}-doped particles. C) The Photoluminescence (PL) spectra of $Eu_{2\%}C7$ and $Eu_{10\%}C7$ NPs, exhibited boarder emission peaks at 590 nm, 610 nm, and 631 nm upon excitation at 397nm.

In-Vitro Analysis:

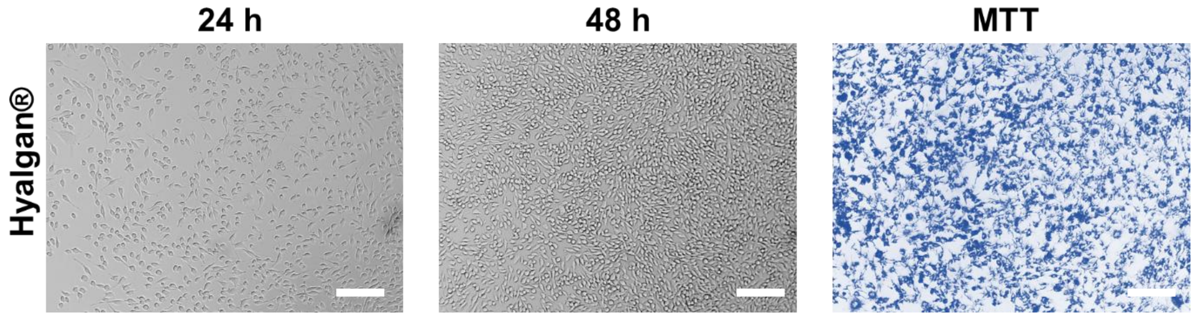

Figure S10. Fibroblast cells treated with control + vehicle (Hyalgan®) after 24 h and 48 h, with MTT staining indicating viable cells. Scale bar= 100 µm

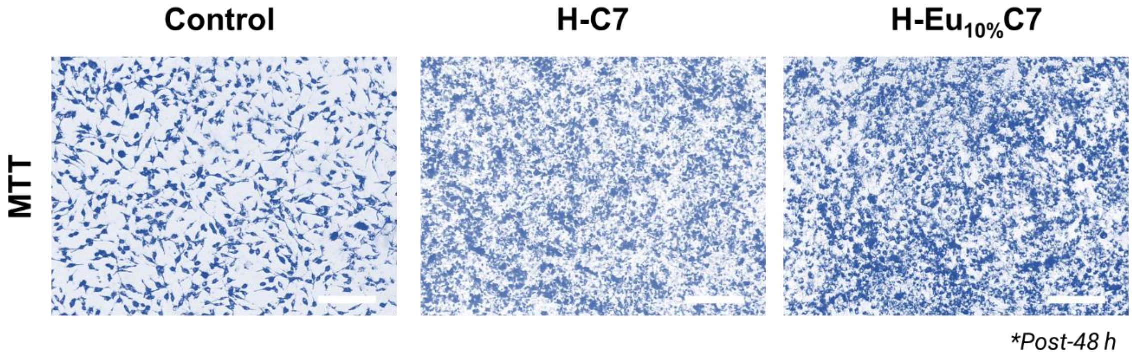

Figure S11. MTT-stained cells after 48 h incubation with CartiOxgel (H-C7 and H-$Eu_{10\%}$C7), showing sustained cell viability following treatment. Scale bar= 100 µm

Histological Examination:

Table S3. Scoring metrics for H&E-stained histological data, evaluating structure, cellularity, Tidemark integrity and matrix integrity to quantify tissue structural changes.

H&E	Group A				Group B			
Criterion	Control		H-Iodine		H-C7		H-Eu$_{10\%}$C7	
	24 h	48 h	24 h	48 h	24 h	48 h	24 h	48 h
Structure	0 (Intact)	0 (Intact)	0 (Intact)	2 (Disrupted)	1 (slight disruption)	1 (Mild Alteration)	0 (Intact)	0 (Intact)
Cellularity	0 (Normal)	0 (Intact)	0 (Intact)	0 (Normal)	0 (Normal)	1 (Minimal Loss)	0 (Normal)	0 (Normal)
Tidemark Integrity	0 (Intact)	0 (Intact)	0 (Intact)	2 (Blurred)	0 (Intact)	1 (Slightly Altered)	0 (Intact)	0 (Intact)
Matrix Integrity	0 (Homogeneous)	0 (Intact)	0 (Intact)	1 (Moderate Loss)	0 (Intact)	0 (Intact)	0 (Homogeneous)	0 (Homogeneous)
**Total H&E Score**	**0**	**0**	**0**	**5**	**1**	**3**	**0**	**0**

Table S4. Scoring metrics for Safranin O-stained histological data, evaluating GAGs content, staining Intensity and homogeneity.

Saf O	Group A				Group B			
Criterion	Control		H-Iodine		H-C7		H-Eu$_{10\%}$C7	
	24 h	48 h	24 h	48 h	24 h	48 h	24 h	48 h
GAGs Content	0 (Normal)	0 (Normal)	0 (Normal)	0 (Normal)	0 (Normal)	1 (Slightly Reduced)	0 (Normal)	0 (Normal)
Staining Intensity	0 (Uniform)	0 (Uniform)	0 (Uniform)	1 (Heterogeneous)	2 (Mildly Reduced)	3 (Reduced Intensity)	0 (Uniform)	1 (Mildly Reduced)
Homogeneity	0 (Intact)	0 (Intact)	0 (Intact)	2 (Disrupted)	0 (Intact)	2 (Partially Altered)	0 (Intact)	0 (Intact)
**Total Saf O Score**	**0**	**0**	**0**	**3**	**2**	**6**	**0**	**1**
